# How women are treated during facility-based childbirth in four countries: a cross-sectional study with labour observations and community-based surveys

**DOI:** 10.1016/S0140-6736(19)31992-0

**Published:** 2019-11-09

**Authors:** Meghan A Bohren, Hedieh Mehrtash, Bukola Fawole, Thae Maung Maung, Mamadou Dioulde Balde, Ernest Maya, Soe Soe Thwin, Adeniyi K Aderoba, Joshua P Vogel, Theresa Azonima Irinyenikan, A Olusoji Adeyanju, Nwe Oo Mon, Kwame Adu-Bonsaffoh, Sihem Landoulsi, Chris Guure, Richard Adanu, Boubacar Alpha Diallo, A Metin Gülmezoglu, Anne-Marie Soumah, Alpha Oumar Sall, Özge Tunçalp

**Affiliations:** a Gender and Women's Health Unit, Centre for Health Equity, Melbourne School of Population and Global Health, University of Melbourne, Carlton, VIC, Australia; b UNDP/UNFPA/UNICEF/WHO/World Bank Special Programme of Research, Development and Research Training in Human Reproduction, Department of Reproductive Health and Research, World Health Organization, Geneva, Switzerland; c Department of Obstetrics and Gynaecology, National Institute of Maternal and Child Health, College of Medicine, University of Ibadan, Ibadan, Nigeria; d Department of Medical Research, Yangon, Myanmar; e Cellule de Recherche en Sante de la Reproduction en Guinee (CERREGUI), Conakry, Guinea; f Department of Population, Family and Reproductive Health, School of Public Health, University of Ghana, Legon, Ghana; g Department of Obstetrics and Gynaecology, Mother and Child Hospital, Oke-Aro, Akure, Ondo State, Nigeria; h Maternal and Child Health Program, Burnet Institute, Melbourne, VIC, Australia; i Department of Obstetrics and Gynaecology, Faculty of Clinical Sciences, University of Medical Sciences, Ondo, Ondo State, Nigeria; j University of Medical Sciences Teaching Hospital, Akure, Ondo State, Nigeria; k Adeoyo Maternity Teaching Hospital, Yemetu, Ibadan, Oyo State, Nigeria; l Department of Obstetrics and Gynaecology, School of Medicine and Dentistry, Unive rsity of Ghana, Accra, Ghana; m Department of Biostatistics, School of Public Health, University of Ghana, Legon-Accra, Ghana; n School of Public Health, University of Ghana, Legon-Accra, Ghana

## Abstract

**Background:**

Women across the world are mistreated during childbirth. We aimed to develop and implement evidence-informed, validated tools to measure mistreatment during childbirth, and report results from a cross-sectional study in four low-income and middle-income countries.

**Methods:**

We prospectively recruited women aged at least 15 years in twelve health facilities (three per country) in Ghana, Guinea, Myanmar, and Nigeria between Sept 19, 2016, and Jan 18, 2018. Continuous observations of labour and childbirth were done from admission up to 2 h post partum. Surveys were administered by interviewers in the community to women up to 8 weeks post partum. Labour observations were not done in Myanmar. Data were collected on sociodemographics, obstetric history, and experiences of mistreatment.

**Findings:**

2016 labour observations and 2672 surveys were done. 838 (41·6%) of 2016 observed women and 945 (35·4%) of 2672 surveyed women experienced physical or verbal abuse, or stigma or discrimination. Physical and verbal abuse peaked 30 min before birth until 15 min after birth (observation). Many women did not consent for episiotomy (observation: 190 [75·1%] of 253; survey: 295 [56·1%] of 526) or caesarean section (observation: 35 [13·4%] of 261; survey: 52 [10·8%] of 483), despite receiving these procedures. 133 (5·0%) of 2672 women or their babies were detained in the facility because they were unable to pay the bill (survey). Younger age (15–19 years) and lack of education were the primary determinants of mistreatment (survey). For example, younger women with no education (odds ratio [OR] 3·6, 95% CI 1·6–8·0) and younger women with some education (OR 1·6, 1·1–2·3) were more likely to experience verbal abuse, compared with older women (≥30 years), adjusting for marital status and parity.

**Interpretation:**

More than a third of women experienced mistreatment and were particularly vulnerable around the time of birth. Women who were younger and less educated were most at risk, suggesting inequalities in how women are treated during childbirth. Understanding drivers and structural dimensions of mistreatment, including gender and social inequalities, is essential to ensure that interventions adequately account for the broader context.

**Funding:**

United States Agency for International Development and the UNDP/UNFPA/UNICEF/WHO/World Bank Special Programme of Research, Development and Research Training in Human Reproduction, Department of Reproductive Health and Research, WHO.

## Introduction

High rates of avoidable maternal and newborn mortality and morbidity in low-income and middle-income countries could be mitigated by improving quality of care.^1^ A *Lancet Global Health* Commission^1^ highlighted the need for high-quality health systems that improve health, and are valued, trusted, and responsive to dynamic population needs. Kruk and colleagues^1^ have called for health systems to “measure and report what matters most to people”, including user experiences, health outcomes, competent care, and confidence in the system. Maternal health indicators have historically focused on process and coverage outcomes related to life-saving interventions (eg, proportion of births with skilled attendance) and health outcomes (eg, maternal mortality). These indicators do not fully reflect or correlate well with quality, nor account for women's perceptions or experiences of care, particularly respect, communication, and emotional support.^2^, ^3^ Poor experiences of maternity care can negatively affect both the woman herself and future health-seeking behaviours,^4^, ^5^ but are typically not routinely assessed. For example, in a Cochrane review of continuous support for women during childbirth, only 11 (41%) of 27 trials reported women's experiences, a primary review outcome.^6^, ^7^

**Research in context**

**Evidence before this study**

The formative phase of our study began in 2014, and before designing our study we did a mixed methods systematic review to synthesise qualitative and quantitative evidence on the mistreatment of women during childbirth in health facilities. We searched all major databases (PubMed, CINAHL, Embase) for relevant studies from inception to Feb 11, 2015, with no date or language restrictions (full methodology including search terms are detailed in a separate publication). Before 2014, only qualitative evidence was available. We identified three measurement studies exploring disrespect and abuse during childbirth as the primary objective. Estimates ranged from 12·2% to 98·0%, and the domains of disrespect and abuse, operational definitions, and measurement approaches (facility exit survey, community-based survey, and labour observations) varied substantially. Since 2015, to our knowledge, seven studies have used labour observations and 15 studies have used women-reported experiences to measure mistreatment during childbirth, but these studies used different periods of interest, measurement tools, and outcomes, thus complicating comparisons. In our 2015 systematic review, we also synthesised evidence from 65 qualitative and quantitative studies done in 34 countries, and developed a typology of what constitutes mistreatment during childbirth. We identified physical and verbal abuse, stigma and discrimination, failure to meet professional standards of care (non-consented procedures and examinations, lack of confidentiality, neglect), poor rapport between women and providers (ineffective communication, lack of supportive care, loss of autonomy), and health system conditions and constraints (resources, policies, and organisational culture) as the primary manifestations experienced or observed during childbirth in health facilities.

**Added value of this study**

This is a multicountry, multisite study of the mistreatment of women during childbirth using two standardised, evidence-informed measurement tools and approaches: continuous observations of women throughout labour, childbirth, and early post-partum periods, and community-based surveys with women at up to 8 weeks post partum. In addition to high frequencies of physical abuse, verbal abuse, and discrimination, we found high frequencies of intervention and non-consented procedures and examinations. For example, 59·0% of observed women and 49·7% of surveyed women did not consent to vaginal examinations, and 75·1% of observed women and 56·1% of surveyed women did not consent to episiotomy. This analysis provides researchers across the world with new tools to measure this important construct. Setting this study in Ghana, Guinea, Myanmar, and Nigeria enabled us to present a cross-sectional view of women's experiences of mistreatment during childbirth in four low-income and middle-income countries across two continents, allowing for comparability of results across multiple domains of mistreatment, ranging from the interpersonal level to the facility level.

**Implications of all the available evidence**

Our study shows that many women experience mistreatment during childbirth, particularly physical and verbal abuse, non-consented care, and detainment. Women were at highest risk of mistreatment during the 30 min before birth until 15 min after birth (observation data). Younger, less educated women were most at risk for mistreatment (survey data), suggesting inequalities in how women are treated during childbirth. Addressing these inequalities and promoting respectful maternity care for all women is essential to improve health equity and quality. Other research in this area has found that mistreatment during childbirth can amount to a violation of human rights, and could be a powerful disincentive from seeking facility-based maternity care. Our study identifies clear gaps in quality and respectful maternity care. Some of these gaps could be addressed through targeted quality improvement initiatives, and others might require addressing structural drivers that perpetuate gender and social inequalities in health care and society more broadly.

Evidence suggests that women across the world experience mistreatment during childbirth, including physical abuse, verbal abuse, discrimination, non-consented procedures, and non-supportive care.^5^ Bowser and Hill's landscape analysis^8^ brought this issue to global attention and our mixed-methods systematic review developed a typology of what constitutes mistreatment.^9^ The WHO intrapartum care guideline recommends respectful maternity care for all women, which is care that maintains “dignity, privacy, and confidentiality, ensures freedom from harm and mistreatment, and enables informed choice and continuous support during labour and childbirth”.^10^ Manifestations and structural drivers of mistreatment are now well documented,^5^, ^11^, ^12^, ^13^ but debate remains about measurement approaches, including the type (observation, woman-reported) and timing (exit interviews, community-based interviews) of measurement.^14^, ^15^, ^16^, ^17^ For example, across 15 studies in seven low-income and middle-income countries,^18^, ^19^, ^20^, ^21^, ^22^, ^23^, ^24^, ^25^, ^26^, ^27^, ^28^, ^29^, ^30^, ^31^, ^32^ location, timing, and populations varied substantially: facility-based exit interviews,^18^, ^19^, ^21^, ^24^, ^26^, ^28^ facility-based interviews during postnatal immunisation,^23^, ^27^ and community-based interviews^20^, ^22^, ^25^, ^26^, ^29^, ^30^, ^31^, ^32^ with women from 3 h to 5 years post partum. The use of different populations, sampling, tools, and data collection methods might influence the risk of bias (selection, social desirability, information, recall) and render cross-study and cross-context comparisons challenging.^14^, ^15^ Accurate measurement is essential to improve accountability, design interventions, and measure impact over time.

In 2013, a technical consultation recommended that WHO initiate research to develop and validate tools to measure the mistreatment of women during childbirth.^17^ The aim of the present study was to use a systematic, evidence-informed approach to develop tools to provide comparable data on the burden of mistreatment across contexts. The formative phase consisted of systematic reviews^9^, ^33^ and primary qualitative research^34^, ^35^, ^36^, ^37^, ^38^, ^39^ in Nigeria, Ghana, Guinea, and Myanmar. Formative research and a review of existing tools informed the measurement phase, which used continuous observations of women during labour and childbirth, and community-based surveys with post-partum women to measure the prevalence of mistreatment in Nigeria, Ghana, Guinea, and Myanmar. We report the prevalence of mistreatment during childbirth based on continuous labour observations and a community-based survey with women.

## Methods

### Study design and participants

Twelve health facilities (maternity hospitals and maternity units within general hospitals [eg, district or regional hospitals]; three per country, all in urban areas) were purposively selected (appendix p 2). Health facilities were included in the study if they were not included in the formative phase, were a secondary-level facility or higher, had at least 200 births per month, had a well defined community catchment area, and allowed non-clinicians to perform observations. Data collection took place in Nigeria from Sept 19, 2016, to Feb 26, 2017, in Ghana from Aug 1, 2017, to Jan 18, 2018, in Guinea from July 1 to Oct 30, 2017, and in Myanmar from Jun 26 to Sept 5, 2017.

The labour observations were continuous, one-to-one observations of women by study researchers from admission, throughout labour and childbirth, until 2 h post partum. Labour observations were not done in Myanmar. The community-based survey was done with women up to 8 weeks post partum.

Women were eligible for the labour observation if they were admitted for childbirth in early established or active labour (<6 cm cervical dilation), were aged at least 15 years, were willing and able to participate, and provided informed consent. Women were not eligible if they were admitted for reasons other than childbirth, immediately transferred or taken directly to theatre, a first-degree relation to a facility employee (mother, sister, cousin), or distressed or otherwise unable to reasonably consent. Pregnant women who were not admitted were eligible to participate if they returned and were admitted for childbirth.

Women were eligible for the survey if they were admitted for childbirth, were aged at least 15 years, were willing and able to participate, resided in the catchment area, and provided consent. Women were not eligible if they were admitted for reasons other than childbirth, were a first-degree relation to a facility employee, were distressed or otherwise unable to reasonably provide consent, resided outside the catchment area, or were unable to provide sufficient contact information.

All women provided written consent. Institutional permission for recruitment and observation was obtained from each site; consent was not sought from providers. This study was approved by the WHO Ethical Review Committee, WHO Review Panel on Research Projects, and in-country ethics committees. The country-specific ethical review committees that reviewed and approved this project were Le Comité National d'Ethique pour la Recherche en Santé (Guinea); Federal Capital Territory Health Research Ethics Committee (Nigeria); Research Ethical Review Committee, Oyo State (Nigeria); State Health Research Ethics Committee of Ondo State (Nigeria); Ethical Review Committee of the Ghana Health Service (Ghana); Ethical and Protocol Review Committee of the College of Health Sciences, University of Ghana (Ghana); and Ethics Review Committee, Department of Medical Research (Myanmar).

### Procedures

Each study site had two or three data collectors per shift to manage recruitment and data collection. All data collectors were experienced women (aged ≥18 years) trained in research methods, and not providers or clinical trainees or students.^14^ All women admitted to the facility during the study period were assessed for eligibility. Data collectors approached women face to face and invited them to participate. Women meeting the eligibility criteria were provided with information about the study and those who agreed to participate consented and were enrolled. Women eligible to participate in both the labour observation and survey were asked to participate in both, and data were linked by the medical record number (results of linked data will be reported elsewhere).

For the labour observations, every eligible woman could not be observed because of practical limitations around the number of data collectors required. To minimise selection bias, when a data collector completed an observation, she returned to the admissions area to enrol the next eligible woman. Each participant was assigned a number, used in all other data collection forms. Further communication between the data collector and the participant was discouraged. The timeframe of interest was from admission until 2 h post partum, facility discharge, or maternal death (whichever happened first). The data collector observed the participant continuously throughout labour, childbirth, and up to 2 h post partum, meaning that there was one data collector per woman, observing only one woman at a time throughout the period of interest. Data collection took place 24 h per day, 7 days per week to ensure no coverage gaps and minimise truncation bias (terminating the observation early because the woman had not given birth). A structured observation guide was used to record interactions between the woman and provider and her birth environment. Recruitment continued until the facility sample size was reached. There was no contact with participants after the observation, unless they were also enrolled in the survey.

For the survey, women received a telephone call at 2–3 weeks post partum to schedule the survey at a time and place of their convenience. Contact was attempted up to three times over 2 weeks. Women who could not be contacted were recorded as lost to follow-up. Data collectors travelled to the interview location, reaffirmed consent, and administered the survey in a private place with no other people present. Recruitment continued until the facility sample size was reached; there was no contact after survey administration.

### Measurements

An iterative mixed-methods approach was used for tool development, described in detail elsewhere.^14^ The typology of mistreatment provided the structure, domains, and items.^5^ Both tools are available in open access.^14^ Data were collected using digital, tablet-based tools (BLU Studio XL2, Android, BLU Products, Miami, FL, USA).

The labour observation tool has three forms completed and submitted at different times: (1) admission form; (2) incident report form; and (3) childbirth, interventions, and discharge form.^14^ The admission form was completed once (immediately after enrolment) for all women, and included screening questions and sociodemographics. The incident report form was completed for the following events: physical or verbal abuse, stigma or discrimination, or vaginal examination, and could be submitted multiple times (repeating form for multiple events). For physical or verbal abuse and stigma or discrimination, the incident report included the timing and type of provider involved. For vaginal examinations, information was collected about consent, privacy, and confidentiality. The childbirth, interventions, and discharge form was completed once at the end of the observation for all women, and included pain relief, mobilisation, fluids, companionship, fees, neglect, privacy, health outcomes, and interventions.

The survey tool had two forms completed and submitted at different times. The screening form assessed eligibility.^14^ The survey form was completed during survey administration,^14^ and included sociodemographics, birth experiences (including mistreatment, vaginal examinations, companionship, and pain relief), health outcomes, interventions, post-partum depression, and satisfaction with care.

### Statistical analysis

Few data exist estimating the prevalence of mistreatment of women during childbirth, complicating sample size calculation. For the labour observation, we prespecified sample size for the development sample (Nigeria) of 130 women per facility and 390 women in total.^14^ For the survey, we used the same calculation and assumed 30% loss to follow-up between recruitment and survey administration; the target sample size for Nigeria was 169 women per facility and 507 women in total. The prevalence of any type of physical abuse, verbal abuse, or stigma or discrimination in Nigeria was used as a proxy for a prevalence estimate for the other countries. For the validation sample, the required sample size was 209 women per facility, and 627 women per country, based on ±5% precision, 80% sensitivity, 5% type 1 error (two-tailed), and 30% prevalence.

Data were submitted using a 3G cellular connection. Consistency checks of screening logs, recruitment, and data were done weekly by WHO and country research teams; inconsistencies were resolved during data collection. Data analysis was done with SAS version 9.4.

For observation and survey data, sociodemographics, health outcomes, and interventions (categorical variables) were aggregated and presented as proportion of the total study population and by country (see appendix p 3 for description of variables).

In the labour observation, specific acts of physical or verbal abuse, and stigma or discrimination were collected as recurring events. Observed events were aggregated and presented as proportion of participants with at least one occurrence for the total study population, stratified by country. We assessed temporal patterns of physical or verbal abuse among women with complete observations for at least 1 h before and after childbirth using two methods: (1) aggregating the total number of incidents in 15-min intervals and deriving a density measure of number of mistreatment events per 1000 women for each 15-min interval, and (2) generalised linear regression to determine odds of an incident in the 15-min interval, relative to 1 h before childbirth, adjusting for country and correlation due to repeated measures.

For the community survey, specific acts of physical or verbal abuse, and stigma or discrimination were aggregated into dichotomous variables (yes or no), then aggregated into a single indicator (yes or no) for each domain. Multivariable logistic regression models were fitted to evaluate factors associated with mistreatment (age, education, marital status, number of previous births, use of curtains) across each domain, adjusting for country. Possible effect modification by woman's education was assessed for verbal abuse, and by single marital status for non-consented vaginal examination. Generalised linear models were fitted to estimate the odds of privacy during vaginal examinations and autonomy, adjusting for a facility clustering effect given potentially different policies across facilities. Outcomes of interest were selected items from each domain: physical abuse; verbal abuse; any physical or verbal abuse, or stigma or discrimination; non-consented vaginal examination; non-private vaginal examination; neglect; long wait times or delays; and autonomy or mobilisation (ie, upright and able to move freely around the room or ward).

### Role of the funding source

All the funders of the study were involved in developing the research question and investigator meetings, but had no other roles in study design, data collection, data analysis, data interpretation, or writing of the report. The corresponding author had full access to all the data in the study and had final responsibility for the decision to submit for publication.

## Results

Results are presented starting with labour observation followed by the survey data, and structured based on the typology of the mistreatment of women during childbirth.^9^ Table 1 shows the sociodemographic and obstetric characteristics by country (see appendix pp 4–5 for additional data).

**Table 1:** Sociodemographic information and obstetric history

	**Labour observation**				**Community survey**				
	Ghana (n=926)	Guinea (n=682)	Nigeria (n=408)	Total (n=2016)	Ghana (n=836)	Guinea (n=644)	Myanmar (n=631)	Nigeria (n=561)	Total (n=2672)
**Maternal age (years)**									
15–19	80 (8·6%)	178 (26·1%)	16 (3·9%)	274 (13·6%)	60 (7·2%)	173 (26·9%)	39 (6·2%)	15 (2·7%)	287 (10·7%)
20–24	167 (18·0%)	207 (30·4%)	63 (15·4%)	437 (21·7%)	148 (17·7%)	186 (28·9%)	167 (26·5%)	74 (13·2%)	575 (21·5%)
25–29	288 (31·1%)	165 (24·2%)	119 (29·2%)	572 (28·4%)	261 (31·2%)	154 (23·9%)	177 (28·1%)	160 (28·5%)	752 (28·1%)
30–34	237 (25·6%)	92 (13·5%)	136 (33·3%)	465 (23·1%)	214 (25·6%)	89 (13·8%)	142 (22·5%)	205 (36·5%)	650 (24·3%)
≥35	154 (16·6%)	40 (5·9%)	74 (18·1%)	268 (13·3%)	153 (18·3%)	42 (6·5%)	106 (16·8%)	107 (19·1%)	408 (15·3%)
**Marital status**									
Single*	153 (16·5%)	34 (5·0%)	18 (4·4%)	205 (10·2%)	134 (16·0%)	45 (6·9%)	18 (2·9%)	34 (6·1%)	231 (8·7%)
Married or cohabitating	740 (79·9%)	634 (93·0%)	382 (93·6%)	1756 (87·1%)	700 (83·7%)	599 (93·0%)	613 (97·2%)	527 (93·9%)	2439 (91·3%)
Other†	33 (3·6%)	14 (2·0%)	8 (1·9%)	55 (2·7%)	2 (0·2%)	0	0	0	2 (0·1%)
**Education**									
No education	49 (5·3%)	318 (46·6%)	4 (1·0%)	371 (18·4%)	35 (4·2%)	286 (44·5%)	15 (2·4%)	2 (0·4%)	338 (12·7%)
Some primary	81 (8·8%)	128 (18·8%)	5 (1·2%)	214 (10·6%)	64 (7·7%)	114 (17·7%)	104 (16·5%)	7 (1·3%)	289 (10·8%)
Some secondary	340 (36·7%)	142 (20·8%)	35 (8·6%)	517 (25·6%)	354 (42·3%)	155 (24·1%)	184 (29·2%)	48 (8·6%)	741 (27·7%)
Complete secondary	304 (32·8%)	47 (6·9%)	175 (42·9%)	526 (26·1%)	257 (30·8%)	45 (7·0%)	193 (30·6%)	237 (42·3%)	732 (27·4%)
Complete tertiary	124 (13·4%)	24 (3·5%)	182 (44·6%)	330 (16·4%)	105 (12·6%)	25 (3·9%)	135 (21·4%)	264 (47·1%)	529 (19·8%)
Vocational or unknown	28 (3·0%)	23 (3·4%)	7 (1·7%)	58 (2·9%)	21 (2·5%)	19 (3·0%)	0	3 (0·6%)	43 (1·6%)
**Number of pregnancies**									
1	245 (26·5%)	232 (34·0%)	115 (28·2%)	592 (29·4%)	231 (27·6%)	209 (32·5%)	325 (51·5%)	156 (27·8%)	921 (34·5%)
2	218 (23·5%)	145 (21·3%)	101 (24·8%)	464 (23·0%)	185 (22·1%)	138 (21·4%)	170 (26·9%)	156 (27·8%)	649 (24·3%)
3	195 (21·1%)	99 (14·5%)	83 (20·3%)	377 (18·7%)	162 (19·4%)	100 (15·5%)	75 (11·9%)	106 (18·9%)	443 (16·6%)
≥4	257 (27·8%)	201 (29·5%)	102 (25·0%)	560 (27·8%)	253 (30·3%)	196 (30·4%)	61 (9·7%)	143 (25·5%)	653 (24·4%)
Other†	11 (1·2%)	5 (0·7%)	7 (1·7%)	23 (1·1%)	5 (0·6%)	1 (0·2%)	0	0	6 (0·2%)
**Number of previous births**									
1	344 (37·1%)	239 (35·0%)	162 (39·7%)	745 (37·0%)	550 (65·8%)	411 (63·8%)	355 (56·3%)	244 (43·5%)	1560 (58·4%)
2	256 (27·6%)	149 (21·8%)	111 (27·2%)	516 (25·6%)	127 (15·2%)	80 (12·4%)	161 (25·5%)	143 (25·5%)	511 (19·1%)
3	320 (34·6%)	292 (42·8%)	134 (32·8%)	746 (37·0%)	70 (8·4%)	56 (8·7%)	67 (10·6%)	85 (15·2%)	278 (10·4%)
≥4	6 (0·6%)	2 (0·3%)	1 (0·2%)	9 (0·4%)	85 (10·2%)	97 (15·1%)	47 (7·5%)	88 (15·7%)	317 (11·9%)
Other†	0	0	0	0	4 (0·5%)	0	1 (0·2%)	1 (0·2%)	6 (0·2%)
**Mode of birth for current (labour observation) or most recent (survey) pregnancy**									
Vaginal birth	760 (82·1%)	558 (81·9%)	362 (88·8%)	1680 (83·3%)	717 (85·8%)	567 (88·0%)	364 (57·7%)	539 (96·1%)	2187 (81·9%)
Caesarean birth	143 (15·4%)	92 (13·5%)	26 (6·4%)	261 (12·9%)	118 (14·1%)	76 (11·8%)	267 (42·3%)	22 (3·9%)	483 (18·1%)
Other†	23 (2·5%)	32 (4·7%)	20 (4·9%)	75 (3·7%)	1 (0·1%)	1 (0·2%)	0	0	2 (0·1%)
**Number of babies at most recent birth**									
1 (singleton)	894 (96·5%)	632 (92·7%)	397 (97·3%)	1923 (95·4%)	818 (97·9%)	626 (97·2%)	621 (98·4%)	552 (98·4%)	2617 (97·9%)
2 (twins)	18 (1·9%)	23 (3·4%)	10 (2·5%)	51 (2·5%)	18 (2·2%)	18 (2·8%)	10 (1·6%)	9 (1·6%)	55 (2·1%)
Other†	14 (1·5%)	27 (4·0%)	1 (0·2%)	42 (2·1%)	14 (1·5%)	2 (0·3%)	0	4 (1·0%)	20 (1·0%)
**Sex of baby at most recent birth**‡									
Female	438 (47·1%)	315 (46·5%)	199 (47·7%)	952 (47·0%)	407 (47·7%)	289 (43·7%)	302 (47·1%)	262 (46·0%)	1260 (46·2%)
Male	485 (52·2%)	361 (53·2%)	218 (52·3%)	1064 (52·5%)	441 (51·6%)	360 (54·4%)	339 (52·9%)	307 (53·9%)	1447 (53·1%)
Unknown	7 (0·8%)	2 (0·3%)	0	9 (0·4%)	6 (0·7%)	13 (2·0%)	0	1 (0·2%)	20 (0·7%)

See appendix (pp 4–5) for additional sociodemographic data.

* Single, separated, divorced, or widowed.

† Other, don't know, unknown, or missing.

‡ Labour observation: 2025 babies; community survey: 2727 babies.

**Figure 1:**
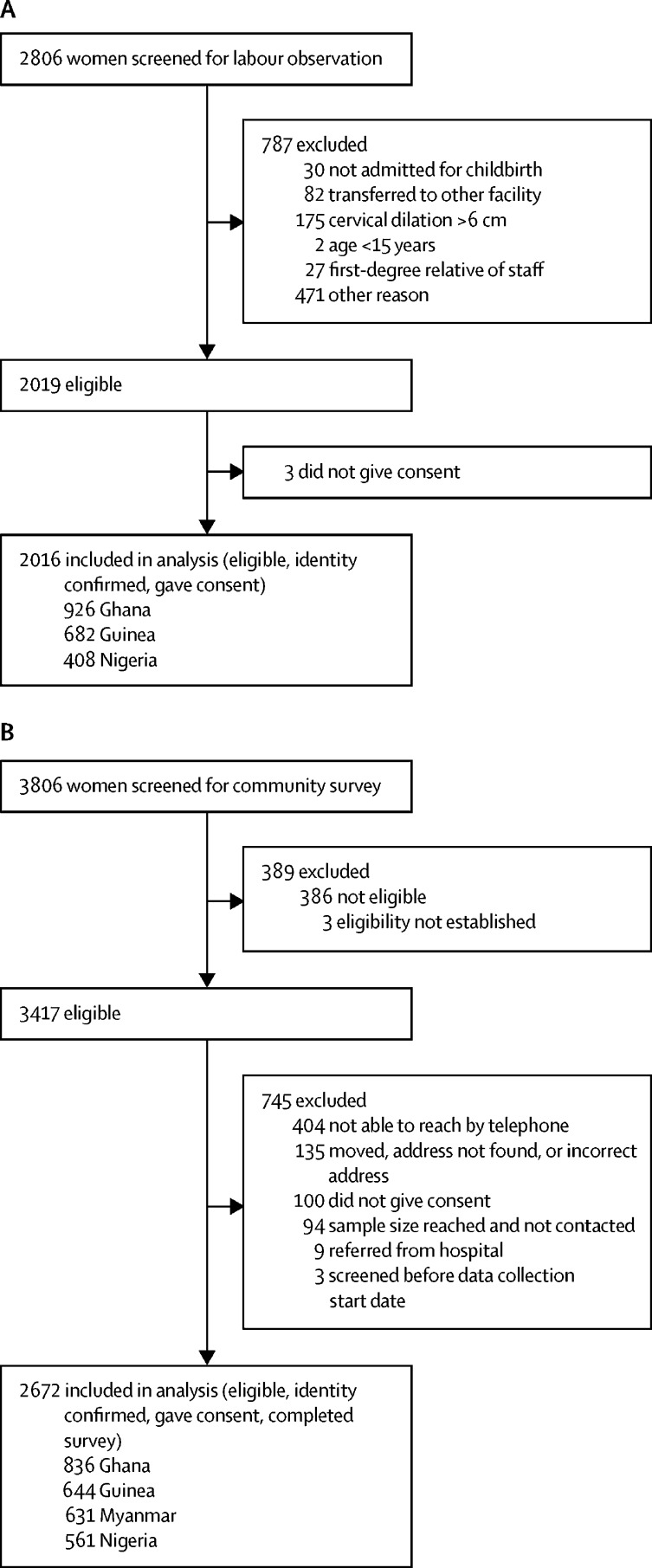
Flow diagram for labour observation (A) and community survey (B)

2016 women were observed (figure 1); the median duration of observation was 5·2 h (IQR 3·8–8·0; Ghana 5·1 h [IQR 3·7–7·9]; Guinea 5·0 h [3·9–7·3]; Nigeria 5·8 h [3·9–9·8]). Observed experiences of mistreatment are shown in table 2 (see appendix pp 6–8 for extended version).

**Table 2:** Mistreatment of women during childbirth (labour observation)

			**Ghana (n=926)**	**Guinea (n=682)**	**Nigeria (n=408)**	**Total (n=2016)**
Any physical abuse, verbal abuse, or stigma or discrimination			293 (31·6%)	269 (39·4%)	276 (67·7%)	838 (41·6%)*
	Any physical abuse		73 (7·9%)	104 (15·2%)	105 (25·7%)	282 (14·0%)*
	Any verbal abuse		272 (29·4%)	228 (33·4%)	262 (64·2%)	762 (37·8%)*
	Any stigma or discrimination		6 (0·7%)	5 (0·7%)	0	11 (0·6%)
Informed consent and confidentiality						
	Caesarean section		143 (15·4%)	92 (13·5%)	26 (6·4%)	261 (12·9%)
		Non-consented	13 (9·1%)	19 (20·7%)	3 (11·5%)	35 (13·4%)
	Episiotomy (1680 women with vaginal birth)†		128 (16·8%)	25 (4·5%)	100 (27·6%)	253 (15·1%)
		Non-consented	96 (75·0%)	22 (88·0%)	72 (72·0%)	190 (75·1%)
Vaginal examinations‡						
	First vaginal examination					
		Did not have any vaginal examination during observation	162 (17·5%)	337 (49·4%)	82 (20·1%)	581 (28·8%)
		Not informed or no permission obtained (1435 women with at least one vaginal examination)§	462 (49·9%)	166 (24·4%)	219 (53·8%)	847 (59·0%)*
	Across all vaginal examinations					
		Total number of vaginal examinations¶	2286 (52·0%)	1051 (23·9%)	1056 (24·0%)	4393 (100·0%)
		Not informed or no permission obtained	1408 (61·6%)	469 (44·6%)	734 (69·5%)	2611 (59·4%)
Pain relief						
	Woman requested pain relief		49 (5·3%)	83 (12·2%)	10 (2·5%)	142 (7·0%)
		Did not receive pain relief	19 (38·8%)	23 (27·7%)	6 (60·0%)	48 (33·8%)
Neglect and abandonment						
	No staff member present when the baby came out (1680 women with vaginal birth)†		17 (2·2%)	43 (7·7%)	15 (4·1%)	75 (4·5%)*
Supportive care						
	Woman not offered to have a labour companion during labour and birth		868 (93·7%)	606 (88·9%)	397 (97·3%)	1871 (92·8%)*
	Companion not present at any time during labour and birth		868 (93·7%)	638 (93·6%)	384 (94·1%)	1890 (93·8%)
	Companion not present at the time of birth		830 (89·6%)	624 (91·5%)	356 (87·3%)	1810 (89·8%)*
	Woman did not have easy access to water or oral fluids during labour (1680 women with vaginal birth)†		326 (42·9%)	164 (29·4%)	162 (44·8%)	652 (38·8%)*
	Woman not told she could mobilise during labour, and did not mobilise during labour		693 (74·8%)	129 (18·9%)	322 (78·9%)	1144 (56·8%)
	Woman not asked for her preferred birthing position		880 (95·0%)	635 (93·1%)	387 (94·9%)	1902 (94·4%)*
Health systems						
	Woman instructed to clean up blood, urine, faeces, or amniotic fluid		0	1 (0·2%)	6 (1·5%)	7 (0·4%)*
	Staff suggested or asked the woman or companion for a bribe, informal payment, or gift		6 (0·6%)	42 (6·2%)	14 (3·4%)	62 (3·1%)*
Curtains, partitions, or other measures used to provide privacy for the woman throughout labour, childbirth, and post-partum periods						
		No	73 (7·9%)	341 (50·0%)	340 (83·3%)	754 (37·4%)*
		Used during some but not all periods	95 (10·3%)	104 (15·3%)	28 (6·9%)	227 (11·3%)*

See appendix (pp 6–8) for extended version.

* Results with significant p values (p<0·05). χ^2^ test used to compare proportions of mistreatment items across countries.

† Number of women with vaginal birth per country: Ghana, n=760; Guinea, n=558; Nigeria, n=362.

‡ Before a vaginal examination, staff informed woman why a vaginal examination was needed and obtained her permission.

§ Number of women with at least one vaginal examination per country: Ghana, n=764; Guinea, n=345; Nigeria, n=326.

¶ The percentages in this row are calculated with the total number of vaginal examinations across the four countries as the denominator (ie, n=4393).

During the labour observations, 838 (41·6%) of 2016 women had observed experiences of physical abuse, verbal abuse, or stigma or discrimination. 282 (14·0%) women experienced physical abuse; most commonly being slapped, hit, or punched (188 [9·3%]), with substantial variation across countries (Guinea 29 [4·3%] of 682 women; Ghana 59 [6·4%] of 926; Nigeria 100 [24·5%] of 408). 63 (3·1%) of 2016 women experienced forceful downward abdominal pressure, and 38 (1·9%) were forcefully held down to the bed. 762 (37·8%) women experienced verbal abuse, with the highest proportion in Nigeria (262 [64·2%] of 408). The most common forms of verbal abuse were being shouted at (548 [27·2%] of 2016 women), scolded (262 [13·0%]), and mocked (162 [8·0%]). 11 (0·6%) women experienced stigma or discrimination, typically regarding their race or ethnicity.

**Figure 2:**
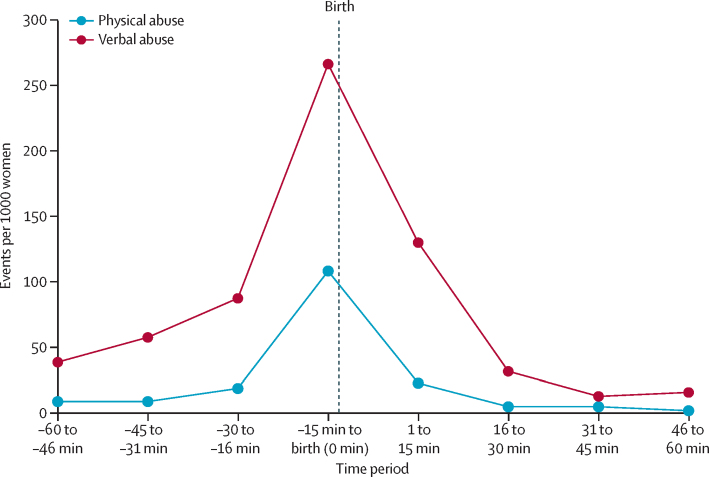
Temporal analysis of mistreatment during labour observation Physical and verbal abuse events per 1000 women. Based on 1590 (78·9%) of 2016 women who were observed for at least 1 h before and after the time of childbirth. Physical and verbal abuse peaked during the period from 30 min before birth until 15 min after birth.

Physical and verbal abuse peaked 30 min before birth until 15 min after birth and were most highly concentrated during the 15-min period before birth (figure 2 and table 3). Women were more likely to be physically abused within 15 min before birth (odds ratio [OR] 11·6, 95% CI 6·2–21·7) and 15 min after birth (OR 2·1, 1·0–4·4), compared with 45–60 min before birth, adjusting for country differences. Similarly, compared with 45–60 min before birth and adjusting for country differences, verbal abuse was elevated 15–30 min before birth (OR 2·2, 95% CI 1·5–3·1), highest within 15 min before birth (OR 6·7, 4·7–9·5), and still elevated within 15 min after birth (OR 3·2, 2·3–4·7).

**Table 3:** Odds of physical and verbal abuse in the time interval relative to 1 h before childbirth

	**Physical abuse**			**Verbal abuse**		
	Events per 1000 women	Odds ratio (95% CI)*	p value	Events per 1000 women	Odds ratio (95% CI)*	p value
46–60 min before birth	8	1 (ref)	..	38	1 (ref)	..
31–45 min before birth	8	0·8 (0·3–2·0)	0·66	57	1·3 (0·9–1·9)	0·19
16–30 min before birth	18	1·5 (0·7–3·1)	0·32	87	2·2 (1·5–3·1)	<0·0001
15 min before and including birth (0 min)	108	11·6 (6·2–21·7)	<0·0001	267	6·7 (4·7–9·5)	<0·0001
1–15 min after birth	22	2·1 (1·0–4·4)	0·044	130	3·2 (2·3–4·7)	<0·0001
16–30 min after birth	4	0·6 (0·3–1·6)	0·32	31	0·8 (0·5–1·3)	0·33
31–45 min after birth	4	0·4 (0·1–1·1)	0·083	12	0·3 (0·1–0·5)	<0·0001
46–60 min after birth	1	0·2 (0·0–0·8)	0·026	15	0·3 (0·1–0·5)	0·0001

Based on 1590 (78·9%) of 2016 women who were observed for at least 1 h before and after the time of childbirth.

* Adjusted for country and potential correlation due to repeated measures.

261 (12·9%) of 2016 women had caesarean birth, of whom 35 (13·4%) did not consent. 253 (15·1%) of 1680 women with vaginal births had episiotomy, most of whom did not consent (190 [75·1%]). Among women with at least one observed vaginal examination (1435 [71·2%] of 2016), at their first vaginal examination 847 (59·0%) of 1435 did not consent. Across all women, 2611 (59·4%) of 4393 vaginal examinations were done without consent.

142 (7·0%) of 2016 women requested pain relief; however, 48 (33·8%) of the 142 women did not receive any. 75 (4·5%) of 1680 women with vaginal births gave birth without a provider present, predominantly in Guinea (43 [7·7%] of 558) and Nigeria (15 [4·1%] of 362).

Most women did not have a companion present (1890 [93·8%] of 2016). Many women did not have access to oral fluids (652 [38·8%] of 1680 women with vaginal birth). Most women in Nigeria and Ghana were not told that they could mobilise and did not mobilise during labour (1015 [76·1%] of 1334), by contrast with Guinea (129 [18·9%] of 682). Most women were not asked their preferred birth position (1902 [94·4%] of 2016); almost all women with non-instrumental vaginal birth used the dorsal, supine, or lithotomy positions (1557 [99·1%] of 1572).

Privacy measures (curtains or partitions) were not used at all for 754 (37·4%) of 2016 women. An additional 227 (11·3%) had privacy measures used inconsistently during labour, childbirth, and post-partum periods. Providers suggested or asked 62 (3·1%) of 2016 women for a bribe or informal payment.

Table 4 shows maternal and newborn health outcomes and interventions (see appendix pp 9–10 for extended version). 278 (13·8%) of 2016 women had induction of labour, 1126 (55·9%) had augmentation of labour, and 125 (6·2%) had perineal shaving. Most women had vaginal birth (1572 [78·0%] of 2016), 108 (5·4%) had instrumental vaginal birth (vacuum or forceps), and 261 (12·9%) had caesarean birth. 118 (5·9%) of 2016 women had a second-degree or higher perineal tear, of whom 113 (95·8%) had perineal repair or suture. 16 (14·2%) of 113 repairs were without local anaesthetic. Within 2 h post partum, 66 (3·3%) of 2016 women were admitted to intensive care, 13 (0·6%) were transferred, 54 (2·7%) discharged, and five (0·2%) women died. Women gave birth to 51 sets of twins (2·5% of women), and 1923 singletons (95·4% of women). Of 2025 babies, there were 50 stillbirths (fresh 31 [1·5%]; macerated 19 [0·9%]) and 44 (2·2%) very early neonatal deaths (within 2 h post partum).

**Table 4:** Maternal and newborn interventions and health outcomes (labour observation)

		**Ghana (n=926)**	**Guinea (n=682)**	**Nigeria (n=408)**	**Total (n=2016)**
**Maternal interventions**					
Induction of labour		237 (25·6%)	25 (3·7%)	16 (3·9%)	278 (13·8%)
Augmentation of labour		529 (57·1%)	401 (58·8%)	196 (48·0%)	1126 (55·9%)
Perineal shaving		82 (8·9%)	7 (1·0%)	36 (8·8%)	125 (6·2%)
Enema		1 (0·1%)	11 (1·6%)	0	12 (0·6%)
Perineal tear					
	First-degree tear	232 (25·1%)	86 (12·6%)	57 (14·0%)	375 (18·6%)
	Second-degree tear	72 (7·8%)	11 (1·6%)	15 (3·7%)	98 (4·9%)
	Third-degree or fourth-degree tear	17 (1·8%)	1 (0·1%)	2 (0·5%)	20 (1·0%)
Perineal repair or suture performed* (n=118)		87 (97·8%)	11 (91·7%)	15 (88·2%)	113 (95·8%)
	Local anaesthetic used during perineal repair (n=113)	78 (89·7%)	10 (90·9%)	9 (60·0%)	97 (85·8%)
**Maternal health outcomes**					
Birth position (for women with non-instrumental vaginal birth, n=1572)					
	Dorsal or supine	295 (42·8%)	519 (97·7%)	314 (89·2%)	1128 (71·8%)
	Lithotomy	384 (55·7%)	8 (1·5%)	37 (10·5%)	429 (27·3%)
	On all fours	0	1 (0·2%)	0	1 (0·1%)
	Squatting or sitting	3 (0·4%)	3 (0·6%)	0	6 (0·4%)
	Lying on her side	6 (0·9%)	0	0	6 (0·4%)
	Other or unknown	1 (0·2%)	0	1 (0·3%)	2 (0·1%)
Mode of childbirth					
	Non-instrumental vaginal birth	689 (74·4%)	531 (77·9%)	352 (86·3%)	1572 (78·0%)
	Instrumental vaginal birth (vacuum or forceps)	71 (7·7%)	27 (4·0%)	10 (2·5%)	108 (5·4%)
	Caesarean section	143 (15·4%)	92 (13·5%)	26 (6·4%)	261 (12·9%)
Maternal admission to intensive care†		36 (3·9%)	14 (2·1%)	16 (3·9%)	66 (3·3%)
Maternal transfer to another hospital†		3 (0·3%)	3 (0·4%)	7 (1·7%)	13 (0·6%)
Maternal discharge†					
	No	908 (98·1%)	604 (88·6%)	390 (95·6%)	1902 (94·3%)
	Yes	0	49 (7·2%)	5 (1·2%)	54 (2·7%)
Maternal status at end of observation†					
	Alive	907 (97·9%)	652 (95·6%)	394 (96·6%)	1953 (96·9%)
	Dead	2 (0·2%)	3 (0·4%)	0	5 (0·2%)
**Newborn health outcomes**					
Singleton or multiple birth					
	Singleton (1 baby)	894 (96·5%)	632 (92·7%)	397 (97·3%)	1923 (95·4%)
	Multiple (set of twins)	18 (1·9%)	23 (3·4%)	10 (2·5%)	51 (2·5%)
	Unknown, don't know, or missing	14 (1·5%)	27 (4·0%)	1 (0·2%)	42 (2·1%)
Sex of the baby (2025 babies)‡					
	Female	438 (47·1%)	315 (46·5%)	199 (47·7%)	952 (47·0%)
	Male	485 (52·2%)	361 (53·2%)	218 (52·3%)	1064 (52·5%)
	Unknown	7 (0·8%)	2 (0·3%)	0	9 (0·4%)
Baby status at birth (2025 babies)‡					
	Baby alive at birth	919 (98·8%)	642 (94·7%)	392 (94·0%)	1953 (96·4%)
	Fresh stillbirth§	3 (0·3%)	24 (3·5%)	4 (1·0%)	31 (1·5%)
	Macerated stillbirth¶	5 (0·5%)	11 (1·6%)	3 (0·7%)	19 (0·9%)
	Unknown, don't know, or missing	3 (0·3%)	1 (0·1%)	18 (4·3%)	22 (1·1%)
Baby status at end of observation period (1953 babies alive at birth)					
	Baby admitted to special care baby unit	124 (13·5%)	14 (2·2%)	53 (13·5%)	191 (9·8%)
	Very early infant death (birth up to 2 h post partum)	15 (1·6%)	17 (2·6%)	12 (3·1%)	44 (2·3%)

See appendix (pp 9–10) for extended version.

* Among women with second, third, or fourth degree perineal tears.

† At up to 2 h post partum (end of observation period).

‡ Number of babies per country: Ghana, n=930; Guinea, n=678; Nigeria, n=417.

§ Stillbirth occurring in the intrapartum period.

¶ Stillbirth probably occurring more than 12 h before birth.

2672 women were interviewed in the community surveys (figure 1). The median duration from date of childbirth to the survey was 43 days (IQR 32–60; Ghana 58 days [IQR 41–78]; Guinea 45 days [31–59]; Myanmar 40 days [32–48]; Nigeria 35 days [21–44]). Self-reported experiences of mistreatment are shown in table 5 (see appendix pp 11–14 for extended version).

**Table 5:** Mistreatment of women during childbirth (community survey)

				**Ghana (n=836)**	**Guinea (n=644)**	**Myanmar (n=631)**	**Nigeria (n=561)**	**Total (n=2672)**
Any physical abuse, verbal abuse, or stigma or discrimination				308 (36·8%)	235 (36·5%)	131 (20·8%)	271 (48·3%)	945 (35·4%)*
	Any physical abuse			52 (6·2%)	124 (19·3%)	21 (3·3%)	90 (16·0%)	287 (10·7%)*
	Any verbal abuse			284 (34·0%)	173 (26·9%)	116 (18·4%)	248 (44·2%)	821 (30·7%)*
	Any stigma or discrimination			31 (3·7%)	9 (1·4%)	11 (1·7%)	28 (5·0%)	79 (3·0%)*
Failure to meet professional standards†								
	Caesarean section			118 (14·1%)	76 (11·8%)	267 (42·3%)	22 (3·9%)	483 (18·1%)
			Non-consented	24 (20·3%)	6 (7·9%)	21 (7·9%)	1 (4·5%)	52 (10·8%)*
	Episiotomy (2187 women with vaginal birth)‡			92 (12·8%)	52 (9·2%)	250 (68·7%)	132 (24·5%)	526 (24·1%)
			Non-consented	38 (41·3%)	38 (73·1%)	166 (66·4%)	53 (40·2%)	295 (56·1%)*
	Induction of labour			125 (15·0%)	3 (0·5%)	173 (27·4%)	48 (8·6%)	349 (13·1%)
			Non-consented	24 (19·2%)	1 (33·3%)	60 (34·7%)	9 (18·8%)	94 (26·9%)
Vaginal examinations								
	Woman had any vaginal examination			808 (96·7%)	589 (91·5%)	505 (80·0%)	543 (96·8%)	2445 (91·5%)
			No consent before vaginal examination	379 (46·9%)	294 (49·9%)	232 (45·9%)	309 (56·9%)	1214 (49·7%)*
			Staff member discussed private health information from vaginal examination so that others could hear	55 (6·8%)	67 (11·4%)	71 (14·1%)	210 (38·7%)	403 (16·5%)
			Vaginal examination not done privately	236 (29·2%)	292 (49·5%)	131 (25·9%)	365 (67·2%)	1024 (41·9%)*
	General description of experience of vaginal examinations							
			Comfortable	71 (8·8%)	153 (26·0%)	375 (74·3%)	46 (8·5%)	645 (26·4%)*
			A little uncomfortable	142 (17·6%)	193 (32·8%)	93 (18·4%)	141 (26·0%)	569 (23·3%)*
			Quite uncomfortable	217 (26·9%)	125 (21·2%)	21 (4·2%)	144 (26·5%)	507 (20·7%)*
			Very uncomfortable	371 (45·9%)	115 (19·5%)	11 (2·2%)	203 (37·4%)	700 (28·6%)*
Pain relief								
	Woman not offered pain relief during time in hospital			511 (61·1%)	456 (70·8%)	90 (14·3%)	471 (83·9%)	1528 (57·2%)*
	Woman requested pain relief			85 (10·2%)	212 (32·9%)	181 (28·7%)	51 (9·1%)	529 (19·8%)
			Woman requested pain relief but did not receive it	40 (47·1%)	67 (31·6%)	15 (8·3%)	29 (56·9%)	151 (28·5%)*
	Woman denied pain relief during time in hospital			46 (5·5%)	19 (3·0%)	34 (5·4%)	50 (8·9%)	149 (5·6%)*
Neglect and abandonment								
	Staff member not present when the baby came out (2187 women with vaginal birth)‡			24 (3·4%)	2 (0·4%)	2 (0·6%)	16 (3·0%)	44 (2·0%)
	Woman waited for long periods of time before attended by health workers			281 (33·6%)	75 (11·7%)	139 (22·0%)	92 (16·4%)	587 (22·0%)*
	Woman felt ignored, neglected, or that presence was a nuisance for health workers or staff			137 (16·4%)	72 (11·2%)	121 (19·2%)	104 (18·5%)	434 (16·2%)*
Communication								
	Language interpretation needed			10 (1·2%)	15 (2·3%)	0	2 (0·4%)	27 (100·0%)
		Interpreter not available		2 (20·0%)	3 (20·0%)	0	2 (100·0%)	7 (25·9%)*
	Woman felt that health workers or staff did not listen and respond to her concerns			105 (12·6%)	113 (17·6%)	200 (31·7%)	59 (10·5%)	477 (17·9%)
Supportive care								
	Not allowed to have a labour companion during labour and birth			486 (58·1%)	336 (52·2%)	5 (0·8%)	373 (66·5%)	1200 (44·9%)*
	Did not have a labour companion present at any point			437 (52·3%)	560 (87·0%)	1 (0·2%)	320 (57·0%)	1318 (49·3%)*
Autonomy								
	Did not have easy access to water or oral fluids (2187 women with vaginal birth)‡			89 (12·4%)	74 (13·1%)	50 (13·7%)	214 (39·7%)	427 (19·5%)*
	Not allowed to eat (2187 women with vaginal birth)‡			298 (41·6%)	82 (14·5%)	1 (0·3%)	327 (60·7%)	708 (32·4%)*
	Woman not told to or did not mobilise during labour			707 (84·6%)	60 (9·3%)	317 (50·2%)	523 (93·2%)	1607 (60·1%)
	Woman did not have a preferred birthing position			810 (96·9%)	517 (80·3%)	512 (81·1%)	526 (93·8%)	2365 (88·5%)
	Woman or baby detained in hospital because of inability to pay hospital bills			40 (4·8%)	56 (8·7%)	7 (1·1%)	30 (5·4%)	133 (5·0%)
Health systems								
	Curtains, partitions, or other privacy measures not used			65 (7·8%)	339 (52·6%)	330 (52·3%)	468 (83·4%)	1202 (45·0%)
	Staff suggested or asked for a bribe, informal payment, or gift			104 (12·4%)	306 (47·5%)	255 (40·4%)	60 (10·7%)	725 (27·1%)
	Woman instructed to clean up own blood, urine, faeces, or amniotic fluid after birth			8 (1·0%)	6 (0·9%)	99 (15·7%)	5 (0·9%)	118 (4·4%)

See appendix (pp 11–14) for extended version.

* Results with significant p values (p<0·05). χ^2^ test used to compare proportions of mistreatment items across countries.

† Procedure explained and woman agreed to the procedure (informed consent).

‡ Number of women with vaginal birth per country: Ghana, n=717; Guinea, n=567; Myanmar, n=364; Nigeria, n=539.

In the community survey, 945 (35·4%) of 2672 women reported physical abuse, verbal abuse, or stigma or discrimination during childbirth, with substantial variation by country (131 [20·8%] of 631 women in Myanmar, 235 [36·5%] of 644 in Guinea, 308 [36·8%] of 836 in Ghana, and 271 [48·3%] of 561 in Nigeria). Across all countries, 287 (10·7%) of 2672 women reported physical abuse, ranging from 21 (3·3%) of 631 in Myanmar to 124 (19·3%) of 644 in Guinea. The most common physical abuse was forceful downward abdominal pressure (158 [5·9%] of 2672 women) and slapping (104 [3·9%]), with substantial between-country variation. 821 (30·7%) of 2672 women reported verbal abuse, with the highest proportions in Nigeria (248 [44·2%] of 561) and Ghana (284 [34·0%] of 836). The most common forms of verbal abuse were being shouted at (533 [20·0%] of 2672 women) and scolding (257 [9·6%]). Women were threatened with poor outcomes for their baby (183 [6·9%] of 2672), particularly in Nigeria (69 [12·3%] of 561) and Ghana (78 [9·3%] of 836). Negative comments about the woman's sexual activity were more common in Nigeria (22 [3·9%] of 561 women) and Ghana (19 [2·3%] of 836) than in Myanmar (six [1·0%] of 631]) and Guinea (five [0·8%] of 644). 79 (3·0%) of 2672 women reported stigma or discrimination, most commonly about their age (28 [1·1%]), economic circumstances (23 [0·9%]), or race or ethnicity (18 [0·7%]).

483 (18·1%) of 2672 women had caesarean birth, of whom 52 (10·8%) did not consent. 526 (24·1%) of 2187 women with vaginal births had episiotomy; most did not consent (295 [56·1%] of 526). 349 (13·1%) of 2672 women had induction of labour, of whom 94 (26·9%) did not consent. Almost half of women who reported at least one vaginal examination did not consent (1214 [49·7%] of 2445), and many reported that the examinations were not done privately (1024 [41·9%] of 2445), particularly in Nigeria (365 [67·2%] of 543), where many women also reported that private health information from the examination was discussed so others could hear (210 [38·7%] of 543). Half of women reported that vaginal examinations were very or quite uncomfortable (1207 [49·4%] of 2445), particularly in Ghana (588 [72·8%] of 808) and Nigeria (347 [63·9%] of 543).

More than half of women (1528 [57·2%] of 2672) were not offered pain relief. 529 (19·8%) of 2672 women requested pain relief but 151 (28·5%) did not receive any, particularly in Nigeria (29 [56·9%] of 51). 44 (2·0%) of 2187 women with vaginal birth gave birth with no provider present. 587 (22·0%) of 2672 women waited for long periods of time before being attended by health workers, particularly in Ghana (281 [33·6%] of 836). 434 (16·4%) of 2652 women reported feeling ignored, neglected, or that their presence was a nuisance for health workers or staff.

477 (17·9%) of 2672 women reported that providers did not listen or respond to their concerns, particularly in Myanmar (200 [31·7%] of 631). Many women (1200 [44·9%] of 2672) were not allowed to have a companion during labour and birth. Of 2187 women with vaginal births, 427 (19·5%) did not have easy access to oral fluids, particularly in Nigeria (214 [39·7%] of 539), and 708 (32·4%) were not allowed food, particularly in Ghana (298 [41·6%] of 717) and Nigeria (327 [60·7%] of 539). Most women in Ghana (707 [84·6%] of 836) and Nigeria (523 [93·2%] of 561) were not told that they could mobilise and did not mobilise during labour, compared with Guinea (60 [9·3%] of 644) and Myanmar (317 [50·2%] of 631). Most women reported no preferred birth position (2365 [88·5%] of 2672). For women with non-instrumental vaginal birth (2187 [81·9%] of 2672), most gave birth in the dorsal, supine, or lithotomy positions (2152 [98·4%] of 2187). 133 (5·0%) of 2672 women or their babies were detained in the facility because they were unable to pay the bill.

Privacy measures (curtains or partitions) were not commonly used (1202 [45·0%] of 2672 women), particularly in Nigeria (468 [83·4%] of 561). Providers suggested or asked 725 (27·1%) of 2672 women for a bribe or informal payment, particularly in Guinea (306 [47·5%] of 644) and Myanmar (255 [40·4%] of 631). Some women were asked to clean up their own blood, urine, faeces, or amniotic fluid after birth (118 [4·4%] of 2672), particularly in Myanmar (99 [15·7%] of 631).

Age was predominantly the single factor associated with different types of mistreatment (table 6). Younger women (15–19 years) were more likely to experience any physical abuse, verbal abuse, or stigma or discrimination (OR 1·9, 95% CI 1·4–2·6), when adjusting for country, education, marital status, and parity. Younger women with no education (OR 3·6, 95% CI 1·6–8·0) and younger women with some education (OR 1·6, 1·1–2·3) were more likely to experience verbal abuse, compared with older women (≥30 years), adjusting for marital status and parity. Younger, unmarried women were more likely to have non-consented vaginal examinations (OR 4·6, 95% CI 1·7–12·3), adjusting for country. Women who reported no use of privacy measures, such as curtains, were more likely to report lack of privacy (OR 3·4, 95% CI 2·3–5·0), compared with women who had privacy measures used, adjusting for age, education, marital status, and parity. Women giving birth for the first time were less likely to report long wait times or delays (OR 0·8, 95% CI 0·6–0·9), compared with women with previous births. Unadjusted and adjusted predictors were not significant for neglect and autonomy outcomes.

**Table 6:** Multivariable logistic regression models to assess factors potentially associated with mistreatment (community survey)

	**Physical abuse**	**Verbal abuse (by no education)**	**Verbal abuse (by at least some education)**	**Any physical or verbal abuse, or stigma or discrimination**	**Non-consented vaginal examination (by single marital status)**	**Non-consented vaginal examination (by marital status other than single)**	**No privacy or lack of privacy during vaginal examination**	**Long wait times or delays**	**Felt ignored, neglected, or their presence was a nuisance**	**Mobilisation**
**Age**										
15–19 years	1·8 (1·1–2·8)*	3·6 (1·6–8·0)†	1·6 (1·1–2·3)‡	1·9 (1·4–2·6)§	4·6 (1·7–12·3)¶	1·2 (0·8–1·6)	1·1 (0·9–1·2)	1·3 (0·9–1·9)	1·0 (0·7–1·6)	1·0 (0·9–1·1)
20–29 years	1·1 (0·8–1·5)	1·1 (0·6–1·9)	1·3 (1·0–1·6)	1·2 (1·0–2·5)	1·9 (0·8–4·9)	1·2 (1·0–1·5)	0·9 (0·9–1·1)	1·1 (0·9–1·4)	1·0 (0·8–1·3)	1·0 (0·9–1·1)
≥30 years	1 (ref)	1 (ref)	1 (ref)	1 (ref)	1 (ref)	1 (ref)	1 (ref)	1 (ref)	1 (ref)	1 (ref)
**Education**										
No education	0·83 (0·6–1·2)	NA	NA	1·0 (0·7–1·3)	NA	NA	1·1 (0·9–1·2)	1·2 (0·8–1·8)	1·2 (0·8–1·8)	NA
At least some education	1 (ref)	NA	NA	1 (ref)	NA	NA	1 (ref)	1 (ref)	1 (ref)	NA
**Marital status**										
Single	1·2 (0·7–1·8)	1·2 (0·4–3·3)	1·1 (0·8–1·6)	1·1 (0·8–1·5)	NA	NA	1·1 (0·9–1·2)	0·8 (0·6–1·2)	0·9 (0·6–1·3)	1·1 (1·0–1·2)
Other than single‖	1 (ref)	1 (ref)	1 (ref)	1 (ref)	NA	NA	1 (ref)	1 (ref)	1 (ref)	1 (ref)
**Number of previous births**										
First birth	1·21 (0·9–1·6)	0·7 (0·4–1·2)	1·0 (0·8–1·2)	1·0 (0·8–1·2)	NA	NA	1·0 (0·9–1·1)	0·8 (0·6–0·9)**	0·9 (0·7–1·1)	1·0 (0·9–1·0)
≥2 births	1 (ref)	1 (ref)	1 (ref)	1 (ref)	NA	NA	1 (ref)	1 (ref)	1 (ref)	1 (ref)
**Curtains used**										
No	NA	NA	NA	NA	NA	NA	3·4 (2·3–5·0)††	NA	NA	NA
Yes	NA	NA	NA	NA	NA	NA	1 (ref)	NA	NA	NA

Data are odds ratio (95% CI). Verbal abuse was stratified by no education and some education because of an interaction between education and age. Non-consented vaginal examinations were stratified by single and not single marital status because of an interaction between age and marital status. Results with significant p values (p<0·05) are indicated. NA=not applicable.

* p=0·0077.

† p=0·0004.

‡ p=0·0460.

§ p=0·0005.

¶ p=0·0014.

‖ Includes currently married, separated, divorced, widowed, cohabitating, or other.

** p=0·0109.

†† p<0·0001.

## Discussion

We report observed experiences of mistreatment from 2016 labour observations, and woman-reported experiences of mistreatment from a community-based survey of 2672 post-partum women. We found that during labour observations, 41·6% of women had experiences of physical abuse, verbal abuse, or stigma or discrimination, most commonly occurring from 30 min before birth until 15 min after birth. The increased risk during this period might be because providers are more likely to be present around the time of birth, or because of stressors influencing provider behaviour (such as availability of resources, and clinical skills to manage childbirth and complications). According to qualitative research, midwives and doctors described women as “uncooperative” during this period and some justified using physical and verbal abuse as “punishment” for non-cooperation and to ensure “good outcomes” for the baby.^34^, ^35^ Although concerns about the baby's wellbeing might provide a partial explanation during this time period in particular, such abusive behaviours will likely only worsen women's anxiety, distress, and disempowerment. Similarly, in the community survey, more than a third of women reported physical abuse, verbal abuse, or stigma or discrimination during labour, with younger, less educated women at highest risk, suggesting inequalities in how women are treated during childbirth. This finding is supported by qualitative research in the study countries showing that adolescents experienced mistreatment because of judgments made by health-care providers about their age and engagement in sexual activity.^38^ Furthermore, observation and survey data show that many women have vaginal examinations and procedures (caesarean section, episiotomy, induction) done without their consent. 4·5% of observed and 2·0% of surveyed women gave birth without the presence of a skilled attendant, and 5·0% of women reported detainment because they were unable to pay the hospital bill.

Other studies exploring mistreatment during childbirth using observations have different periods of interest, clinical observers, measurement tools, and outcomes, thus complicating comparison (appendix pp 15–16).^18^, ^21^, ^30^, ^40^, ^41^, ^42^, ^43^ In brief, studies in Ethiopia, Malawi, and Kenya reported lower frequencies of physical abuse (0·2% to 9·0%) and verbal abuse (1·9% to 18·1%),^40^, ^41^, ^43^ and similar proportions of non-consented procedures (17·1% to 77·0%)^41^, ^43^ and non-consented vaginal examinations (20·5% to 81·0%).^41^, ^42^ Four studies measured mistreatment using community-based surveys within a similar time period (2–12 weeks post partum).^20^, ^24^, ^25^, ^30^ Studies in India, Brazil, and Tanzania showed lower frequencies of physical abuse (<1·0% to 5·1%) and verbal abuse (2·6% to 10·0%). Afulani and colleagues^44^ measured person-centred care in Ghana, India, and Kenya, and reported that providers did not explain the purpose of examinations or procedures for two-thirds of women, slightly higher than the proportion seen in our study.

Key strengths of our study are the use of an evidence-informed typology of mistreatment, and measurement tools based on an iterative development process including primary qualitative research. For the observations, we had 24 h per day, 7 days per week data collection, reducing the risk of selection and truncation bias. Non-clinical observers might reduce the risk of under-reporting because of the normalisation of mistreatment by clinical observers. Non-clinical observers might have difficulty understanding clinical aspects of childbirth; we addressed this issue by including obstetricians and midwives in training workshops. Our observations might be limited by the Hawthorne effect because the observers' presence might alter provider behaviour, resulting in underestimation of mistreatment. Exploration of the Hawthorne effect by facility, country, and month of recruitment in our study showed no evidence of its presence (appendix pp 17–18). In the survey, we prospectively identified all eligible women, and had lower than expected loss to follow-up (548 [16·0%] of 3417 eligible women). Surveys were community-based to reduce the risk of bias inherent with exit interviews (courtesy or social desirability). We asked women about experiences of specific items of mistreatment^9^ rather than an overall question about experience of mistreatment to reduce information bias, which is in agreement with standards of measuring violence against women.^45^ Some experiences of mistreatment are more subjective (eg, discrimination); future analyses are planned on a linked subgroup who participated in both the observation and survey to explore subjectivity of experiences. The median time from birth to survey administration varied from 35 days (IQR 21–44) in Nigeria to 58 days (41–78) in Ghana, which might have affected recall. Finally, all study facilities were public and in urban areas, which might limit generalisability. Despite the risk of under-estimation inherent in the use of observational and self-reported data collection methods, we found that mistreatment during childbirth was a serious issue, and use of two separate measurement methods is a key strength of this study.

More work is needed to explore how woman-reported experiences of mistreatment during childbirth can be feasibly integrated into quality improvement initiatives, and further adaptation will be required to use these tools for facility-based assessments of women's experiences within widely used mechanisms.^46^ Further analyses are in progress to develop consolidated measurement scales, which might be more practical for targeted monitoring and quality improvement. Within country implementation, the Quality of Care Network is standardising measurement of women's maternity care experiences, and nine low-income and middle-income countries are currently testing these tracer indicators.^1^, ^47^

Understanding drivers and structural dimensions of mistreatment during childbirth, such as gender and social inequalities, and judgments about women's sexuality, is essential to ensure that any interventions adequately account for societal context. Sen and colleagues^12^ hypothesised that structural dimensions influence mistreatment during childbirth via historical biases, power inequalities, normalisation of poor treatment, and communication barriers. Further research is needed to understand how institutional structures and processes can be reorganised to provide better woman-centred care. The depth of these challenges suggests that it is unlikely that interventions that do not address these factors, such as one-off training, will have a lasting effect on behaviour change. This situation is further complicated by the fact that many providers, particularly midwives, are women and experience mistreatment and gender discrimination themselves both within and outside of the health system.^48^

Nevertheless, our study shows clear areas for targeted quality improvement that addresses country-specific or facility-specific challenges, or both, particularly around communication and consent. Provider training to support women to give birth in other positions might build the confidence of providers. Mobilising and upright positions in the first stage of labour might reduce duration of labour and caesarean birth,^49^ and are recommended by WHO.^10^ Addressing some areas of mistreatment (eg, privacy, companionship, and pain relief) might require structural changes—for example cheap but effective measures such as curtains—at a facility or system level. Further research is needed to explore the effect of labour companionship on mistreatment, but the presence of a companion, another recommendation by WHO,^10^ has a positive effect on outcomes including women's birth experiences.^6^, ^7^, ^50^ Civil society and community groups should continue to advocate for respectful care for all women, and empower people to hold health systems accountable. Ultimately, to achieve respectful maternity care, the balance of power must shift from systems to people, and to women themselves.

In conclusion, more than 40% of observed women and 35% of surveyed women experienced mistreatment during childbirth. Younger, less educated women were at highest risk, highlighting the need for multilevel interventions. Addressing these inequalities and promoting respectful maternity care for all are key to improve health equity and quality. Our findings can be used to inform policies and programmes to ensure that all women have positive pregnancy and childbirth experiences, and are supported by empowered health-care providers within well functioning health systems. Action is urgently needed to enhance the provision of respectful maternity care worldwide.
