# Supplementary material for: How women are treated during facility-based childbirth in four countries: a cross-sectional study with labour observations and community-based surveys
Source: Lancet. 2019 Nov 9;394(10210):1750–63. doi: 10.1016/S0140-6736(19)31992-0 (PMC6853169; doi:10.1016/S0140-6736(19)31992-0)
Supplement: Supplementary appendix [file mmc1.pdf]

THE LANCET

Supplementary appendix

This appendix formed part of the original submission and has been peer reviewed. We post it as supplied by the authors.

Supplement to: Bohren MA, Mehrtash H, Fawole B, et al. How women are treated during facility-based childbirth in four countries: a cross-sectional study with labour observations and community-based surveys. *Lancet* 2019; published online Oct 8. http://dx.doi.org/10.1016/S0140-6736(19)31992-0.

Web appendices

Page 2. Facility characteristics table

Page 3. Description of variables.

Page 4. **Labor observation and community survey**: additional sociodemographic information

Page 6. **Labor observation**: Physical abuse, verbal abuse, and stigma and discrimination

Page 7. **Labor observation**: Failure to meet professional standards of care, poor rapport between women and providers, health systems

Page 9. **Labor observation**: Maternal and newborn interventions and health outcomes

Page 11. **Community survey**: Physical abuse, verbal abuse, and stigma and discrimination

Page 12. **Community survey**: Failure to meet professional standards of care, poor rapport between women and providers, health systems

Page 15. Comparison of our study to other mistreatment studies using labor observation and community-based surveys

Page 17. **Labor observation**: Assessing the presence of the Hawthorne effect, by country and month of recruitment, based on any experience of physical abuse, verbal abuse, or stigma and discrimination.

Web appendices

Facility characteristics table

	Ghana			Guinea			Myanmar			Nigeria		
	**Facility 1**	**Facility 2**	**Facility 3**	**Facility 4**	**Facility 5**	**Facility 6**	**Facility 7**	**Facility 8**	**Facility 9**	**Facility 10**	**Facility 11**	**Facility 12**
**Location**	Urban	Urban	Urban	Urban	Urban	Urban	Urban	Urban	Urban	Urban	Urban	Urban
**Management**	Public	Public	Public	Public	Public	Public	Public	Public	Public	Public	Public	Public
**Cost of vaginal birth (USD)**	$51-310, depending on insurance coverage	$41-186, depending on insurance coverage	$0-41, depending on insurance coverage	Free	Free	Free	Free	Free	Free	$12	Free	Free
**Cost of caesarean birth (USD)**	$206-517 depending on insurance	$206-413, depending on insurance	$0-206, depending on insurance	Free	Free	Free	Free	Free	Free	$218-328	Free	Free
**Total births per month**	790	560	251	207	217	375	1506	551	305	160	450	200
**Number of beds - obstetrics**	261	113	56	15	20	27	270	100	100	90	53	57
**Caesarean section rate**	46%	48%	31%	7%	9%	12%	40%	30%	31%	25%	20%	34%
**# obstetricians per shift**	9	1	1	0	0	6	13	3	1	1	3 (day), 1 (night)	1
**# medical officers/junior doctors per shift**	10	7	2	5	4	4	1	2	1	3	8 (day), 2 (night)	1
**# midwives per shift**	19	10	7	2	2	2	10	2	1	6	12 (day), 5 (night)	1
**Estimated population of the catchment area**	315,051*	148,903*	216,605	280,817	519,491	163,481	*	*	*	1,500,000	750,000	800,000
**Facility-based childbirth in catchment area**	92%	92%	92%	39%	35%	43%	69%**	69%**	69%**	75%	60%	60%

* Catchment area includes the whole city, not limited to specific neighborhoods
** Rate in the metropolitan area

Web appendices

Description of Variables

In the labor observation, internal validity of the subsample was evaluated by comparing the pattern of abuse one-hour before and after childbirth in two subgroups: the first group of participants for whom labor observation started ≥2-hours before birth, and second group of participants for whom labor observation started <2-hours but >1-hour prior to childbirth.

For both the labor observation and community survey, we developed a few composite indicators. We developed composite indicators to measure the occurrence of the informed consent process (provider explaining the procedure to the woman and the woman agreeing to the procedure) for caesarean section, episiotomy, induction of labor and vaginal examinations. Mobilization was coded as whether a woman was told to mobilize during labor or did mobilize.

In the community survey, we developed a composite indicator for neglect (feeling neglected, like a nuisance, or ignored). Communication was coded as listening to or responding a woman's concerns.

Differences between countries at $p<0.01$ significance-level were calculated.

Web appendices

Labor observation and community survey: additional sociodemographic information

Labor observation								
	**Ghana**		**Guinea**		**Myanmar**		**Total**	
	n	%	n	%	n	%	n	%
**Overall sample**	926	45·9	682	33·8	408	20·2	2016	100·0
**Number of previous abortions**								
0	669	72·2	623	91·3	326	79·9	1618	80·3
1	248	26·8	57	8·4	79	19·4	384	19·0
Unknown/don't know	9	1·0	2	0·3	3	0·7	14	0·7
**Number of previous miscarriages**								
0	792	85·5	659	96·6	328	80·4	1779	88·2
≥1	120	13·0	21	3·1	76	18·6	217	10·8
Unknown/don't know	14	1·5	2	0·3	4	1·0	20	1·0
**HIV status**								
HIV negative	889	96·0	421	61·7	385	94·4	1695	84·1
HIV positive	17	1·8	8	1·2	9	2·2	34	1·7
Unknown/don't know	20	2·2	253	37·1	14	3·4	287	14·2
**Mode of birth for current (labor observation)**								
Non-instrumental vaginal birth	689	74·4	531	77·9	352	86·3	1572	78·0
Instrumental vaginal birth (vacuum/forceps)	71	7·7	27	4·0	10	2·5	108	5·4
Caesarean birth	143	15·4	92	13·5	26	6·4	261	12·9
Other*	23	2·5	32	4·7	20	4·9	75	3·7

Web appendices

Community survey										
	**Ghana**		**Guinea**		**Myanmar**		**Nigeria**		**Total**	
	n	%	n	%	n	%	n	%	n	%
**Overall sample**	836	31·3	644	24·1	631	23·6	561	21·0	2672	100·0
**Number of children alive today**										
0	7	0·8	17	2·6	2	0·3	7	1·3	33	1·2
1	319	38·2	227	35·3	358	56·7	203	36·2	1107	41·4
2	218	26·1	134	20·8	162	25·7	174	31·0	688	25·8
3	140	16·8	107	16·6	60	9·5	92	16·4	399	14·9
≥ 4	148	17·7	159	24·7	49	7·8	85	15·2	441	16·5
Unknown/don't know	4	0·5	0	0·0	0	0·0	0	0·0	4	0·2
**Currently breastfeeding**										
No	18	2·2	46	7·1	14	2·2	9	1·6	87	3·3
Yes	818	97·9	598	92·9	616	97·6	551	98·2	2583	96·7
**Breastfeeding initiation**										
< 1 hour	407	48·7	271	42·1	444	70·4	218	38·9	1340	50·2
> 1 hour < 24 hours	326	39·0	308	47·8	121	19·2	253	45·1	1008	37·7
> 24 hours	88	10·5	26	4·0	56	8·9	83	14·8	253	9·5
Unknown/missing	15	1·8	39	6·1	10	1·6	7	1·3	71	2·7
**Baby Status**										
Baby alive at interview	830	97·2	612	92·5	634	98·9	561	98·4	2637	96·7
Stillbirth	11	1·3	29	4·4	1	0·2	1	0·2	42	1·5
Very early neonatal death (between birth and discharge)	4	0·5	11	1·7	5	0·8	6	1·1	26	1·0
Neonatal death (between discharge and interview)	7	0·8	10	1·5	0	0·0	2	0·4	19	0·7
Unknown	2	0·2	0	0·0	1	0·2	0	0·0	3	0·1

Web appendices

Labor observation: Physical abuse, verbal abuse, and stigma and discrimination

	Ghana		Guinea		Nigeria		Total	
	n	%	n	%	n	%	n	%
Overall sample	926	45·9	682	33·8	408	20·2	2016	100·0
**Any physical abuse, verbal abuse, or stigma and discrimination**	293	31·6	269	39·4	276	67·7	838	41·6
**Any physical abuse**	73	7·9	104	15·2	105	25·7	282	14·0
Slap	58	6·3	1	0·1	94	23·0	153	7·6
Forceful downward pressure on abdomen	4	0·4	50	7·3	9	2·2	63	3·1
Held down to the bed forcefully	12	1·3	17	2·5	9	2·2	38	1·9
Punch	1	0·1	23	3·4	4	1	28	1·4
Hit	0	0	5	0·7	2	0·5	7	0·4
Kick	1	0·1	1	0·1	3	0·7	5	0·3
Pinch	2	0·2	1	0·1	0	0	3	0·2
Gag	2	0·2	1	0·1	0	0	3	0·2
Tied to the bed	0	0	0	0	0	0	0	0·0
Other physical abuse	9	1	27	4	6	1·5	42	2·1
**Any verbal abuse**	272	29·4	228	33·4	262	64·2	762	37·8
Shouted at	190	20·5	144	21·1	214	52·5	548	27·2
Scolded	75	8·1	81	11·9	106	26·0	262	13·0
Mocked	22	2·4	52	7·6	88	21·6	162	8·0
Insulted	25	2·7	17	2·5	82	20·1	124	6·2
Threatened with poor outcome for baby	27	2·9	14	2·1	33	8·1	74	3·7
Hissed at	17	1·8	15	2·2	33	8·1	65	3·2
Negative comments - her sexual activity	33	3·6	5	0·7	22	5·4	60	3·0
Threatened with medical procedure	19	2·1	5	0·7	17	4·2	41	2·0
Negative comments - her appearance	15	1·6	4	0·6	21	5·1	40	2·0
Threatened to withhold care	13	1·4	2	0·3	18	4·4	33	1·6
Negative comments - baby's appearance	5	0·5	8	1·2	7	1·7	20	1·0
Threatened with physical violence	5	0·5	7	1	7	1·7	19	0·9
Blamed woman for poor outcome	3	0·3	1	0·1	5	1·2	9	0·4
**Any stigma and discrimination**	6	0·7	5	0·7	0	0	11	0·6
Race/ethnicity	1	0·1	4	0·6	0	0	5	0·3
Economic circumstances	2	0·2	0	0	0	0	2	0·1
Age	1	0·1	0	0	0	0	1	0·1
Marital status	0	0	1	0·1	0	0	1	0·1
Level of education/literacy	0	0	0	0	0	0	0	0·0
Religion	0	0	0	0	0	0	0	0·0
HIV status	0	0	0	0	0	0	0	0·0
Other stigma/discrimination	2	0·2	1	0·1	0	0	3	0·2

Web appendices

Labor observation: Failure to meet professional standards of care, poor rapport between women and providers, health systems

	Ghana		Guinea		Nigeria		Total	
	n	%	n	%	n	%	n	%
Overall sample	926	45·9	682	33·8	408	20·2	2016	100·0
Informed consent and confidentiality								
Procedure explained and woman agreed to the procedure								
**Caesarean section (n=261)**	143	15·4	92	13·5	26	6·4	261	12·9
Not explained, did not agree	0	0·0	9	9·8	0	0·0	9	3·5
Not explained, agreed	13	9·1	9	9·8	3	11·5	25	9·6
Explained, did not agree	0	0·0	1	1·1	0	0·0	1	0·4
Explained and agreed	125	87·4	73	79·4	23	88·5	221	84·7
Don't know/refuse/missing	5	3·5	0	0·0	0	0·0	5	2·0
**Episiotomy (n=1680 women with vaginal birth)**	128	16·8	25	4·5	100	27·6	253	15·1
Not explained, did not agree	75	58·6	20	80·0	57	55·3	151	59·7
Not explained, agreed	10	7·8	2	8·0	8	7·8	20	7·9
Explained, did not agree	11	8·6	0	0·0	8	8·0	19	7·5
Explained and agreed	29	22·7	3	12·0	24	24·0	56	22·1
Don't know/refuse	3	2·3	0	0·0	4	3·9	7	2·7
Vaginal exams								
Before a vaginal examination, staff informed woman why a vaginal exam was needed and obtained her permission								
**First vaginal exam**								
Did not have any vaginal exam during observation period	162	17·5	337	49·4	82	20·1	581	28·8
Not informed, permission not obtained	215	23·2	105	15·4	92	22·6	412	28·7
Not informed, permission obtained	216	23·3	55	8·1	77	18·9	348	24·3
Informed, permission not obtained	31	3·4	6	0·9	50	12·3	87	6·1
Informed and permission obtained	300	32·4	177	26·0	104	25·5	581	40·5
Don't know/refuse	2	0·2	2	0·3	3	0·7	7	0·5
**Across all vaginal exams**								
Total # of vaginal exams conducted	2286	52·0	1051	23·9	1056	24·0	4393	-
Not informed, permission not obtained	632	27·6	294	28·0	305	28·9	1231	28·0
Not informed, permission obtained	676	29·6	155	14·7	280	26·5	1111	25·3
Informed, permission not obtained	100	4·4	20	1·9	149	14·1	269	6·1
Informed and permission obtained	878	38·4	582	55·4	322	30·5	1782	40·6
**Pain relief**								
**Woman requested and received pain relief**								
Not requested, not received	877	94·7	599	87·8	398	97·6	1874	93·0
Requested, not received	19	2·1	23	3·4	6	1·5	48	2·4
Requested and received	30	3·2	60	8·8	4	1·0	94	4·7
**Neglect and abandonment**								
**Staff member present when the baby came out (n=1680 women with vaginal birth)**								
No	17	2·2	43	7·7	15	4·1	75*	4·5
Yes	743	97·8	515	92·3	347	95·9	1605	92·9
Missing	0	0	0	0	0	0	0	0
**Supportive care**								
Woman offered to have a labor companion during labor and birth								
No	868	93·7	606	88·9	397	97·3	1871	92·8
Yes	58	6·3	75	11·0	11	2·7	144	7·1
Missing	0	0·0	1	0·2	0	0·0	1	0·1
**Companion present at any time during labor and birth**								
No	868	93·7	638	93·6	384	94·1	1890	93·8
Yes	58	6·3	44	6·5	24	5·9	126	6·3
**Companion present at the time of birth**								
No	830	89·6	624	91·5	356	87·3	1810	89·8
Yes	82	8·9	31	4·6	51	12·5	164	8·1
Missing	14	1·5	27	4·0	1	0·3	42	2·1
**Autonomy**								

**Woman had easy access to water or oral fluids during labor (n=1680 women with vaginal birth)**								
No	326	42·9	164	29·4	162	44·8	652*	38·8
Yes	434	57·1	393	70·4	199	54·9	1026	61·1
Unknown/don't know	0	0·0	1	0·2	1	0·3	2	0·1
**Woman told she could mobilize during labor, and if she mobilized during labor**								
Not told, did not mobilize	693	74·8	129	18·9	322	78·9	1144	56·8
Told, did not mobilize	16	1·7	11	1·6	2	0·5	29	1·4
Not told, mobilized	13	1·4	23	3·4	62	15·2	98	4·9
Told and mobilized	204	22·0	519	76·1	21	5·2	744	36·9
Don't know/refuse	0	0·0	0	0·0	1	0·3	1	0·1
**Woman asked for her preferred birthing position**								
No	880	95·0	635	93·0	387	94·9	1902	94·4
Yes	14	1·5	14	2·1	0	0·0	28	1·4
Unknown/don't know	32	3·5	33	4·8	21	5·2	86	4·3
**Birth position (for women with non-instrumental vaginal birth, n=1572)**								
Dorsal/supine	295	42·8	519	97·7	314	89·2	1128	71·8
Lithotomy	384	55·7	8	1·5	37	10·5	429	27·3
On all fours	0	0·0	1	0·2	0	0·0	1	0·1
Squatting or sitting	3	0·4	3	0·6	0	0·0	6	0·4
Lying on her side	6	0·9	0	0·0	0	0·0	6	0·4
Other/unknown	1	0·2	0	0·0	1	0·3	2	0·1
**Woman instructed to clean up blood, urine, feces or amniotic fluid**								
No	926	100·0	681	99·9	402	98·5	2009	99·7
Yes	0	0·0	1	0·2	6	1·5	7	0·4
**Staff suggested or asked the woman or companion for a bribe, informal payment, or gift**								
No	920	99·4	640	93·8	394	96·6	1954	96·9
Yes	6	0·6	42	6·2	14	3·4	62	3·1
**Curtains, partitions, or other measures used to provide privacy for the woman throughout labor, childbirth and postpartum periods**								
Yes	755	81·5	237	34·8	26	6·4	1018	50·5
No	73	7·9	341	50·0	340	83·3	754	37·4
Used during some but not all periods	95	10·3	104	15·3	28	6·9	227	11·3
Unknown/don't know	3	0·3	0	0	14	3·4	17	0·8

* Was the procedure explained and did the woman agree to the procedure?
** Before a vaginal examination, did staff inform woman why a vaginal exam was needed and obtain permission?

Web appendices

Labor observation: Maternal and newborn interventions and health outcomes

	Ghana		Guinea		Nigeria		Total	
	n	%	n	%	n	%	n	%
Maternal interventions								
**Induction of labor**								
No	669	72·2	602	88·3	375	91·9	1646	81·6
Yes	237	25·6	25	3·7	16	3·9	278	13·8
Unknown/don't know/missing	20	2·2	55	8·1	17	4·2	92	4·6
**Augmentation of labor**								
No	373	40·3	252	37·0	198	48·5	823	40·8
Yes	529	57·1	401	58·8	196	48·0	1126	55·9
Unknown/don't know/missing	24	2·6	29	4·3	14	3·4	67	3·3
**Perineal shaving**								
No	821	88·7	645	94·6	356	87·3	1822	90·4
Yes	82	8·9	7	1·0	36	8·8	125	6·2
Unknown/don't know/missing	23	2·5	30	4·4	16	3·9	69	3·4
**Enema**								
No	901	97·3	641	94·0	391	95·8	1933	95·9
Yes	1	0·1	11	1·6	0	0·0	12	0·6
Unknown/don't know/missing	24	2·6	30	4·4	17	4·2	71	3·5
**Perineal tear**								
No tear	560	60·5	545	79·9	304	74·5	1409	69·9
First degree tear	232	25·1	86	12·6	57	14·0	375	18·6
Second degree tear	72	7·8	11	1·6	15	3·7	98	4·9
Third- or fourth-degree tear	17	1·8	1	0·1	2	0·5	20	1·0
Unknown/don't know/missing	45	4·9	39	5·7	30	7·4	114	5·7
**Perineal repair or suture performed (n=118)**								
No	1	1·1	1	8·3	2	11·8	4	3·4
Yes	87	97·8	11	91·7	15	88·2	113	95·8
Unknown/don't know/missing	1	1·1	0	0·0	0	0·0	1	0·8
**Local anesthetic used during perineal repair (n=113)**								
No	9	10·3	1	9·1	6	40·0	16	14·2
Yes	78	89·7	10	90·9	9	60·0	97	85·8
Maternal health outcomes								
**Birth position (for women with non-instrumental vaginal birth, n=1572)**								
Dorsal/supine	295	42·8	519	97·7	314	89·2	1128	71·8
Lithotomy	384	55·7	8	1·5	37	10·5	429	27·3
On all fours	0	0·0	1	0·2	0	0·0	1	0·1
Squatting or sitting	3	0·4	3	0·6	0	0·0	6	0·4
Lying on her side	6	0·9	0	0·0	0	0·0	6	0·4
Other/unknown	1	0·2	0	0·0	1	0·3	2	0·1
**Mode of childbirth**								
Non-instrumental vaginal birth	689	74·4	531	77·9	352	86·3	1572	78·0
Instrumental vaginal birth (vacuum/forceps)	71	7·7	27	4·0	10	2·5	108	5·4
Caesarean section	143	15·4	92	13·5	26	6·4	261	12·9
Other/unknown/missing	23	2·5	32	4·7	20	4·9	75	3·7
**Maternal admission to intensive care***								
No	870	94·0	638	93·5	373	91·4	1881	93·3
Yes	36	3·9	14	2·1	16	3·9	66	3·3
Unknown/don't know/missing	20	2·2	30	4·4	19	4·7	69	3·4
**Maternal transfer to another hospital***								
No	901	97·3	650	95·3	386	94·6	1937	96·1
Yes	3	0·3	3	0·4	7	1·7	13	0·6
Unknown/don't know/missing	22	2·4	29	4·3	15	3·7	66	3·3
**Maternal discharge***								
No	908	98·1	604	88·6	390	95·6	1902	94·3
Yes	0	0·0	49	7·2	5	1·2	54	2·7
Unknown/don't know/missing	18	1·9	29	4·3	13	3·2	60	3·0
**Maternal status at end of observation***								
Alive	907	97·9	652	95·6	394	96·6	1953	96·9
Dead	2	0·2	3	0·4	0	0·0	5	0·2
Unknown/don't know/missing	17	1·8	27	4·0	14	3·4	58	2·9
Newborn health outcomes								

**Singleton or multiple birth**								
Singleton (1 baby)	894	96·5	632	92·7	397	97·3	1923	95·4
Multiple (set of twins)	18	1·9	23	3·4	10	2·5	51	2·5
Unknown/don't know/missing	14	1·5	27	4·0	1	0·2	42	2·1
**Sex of the baby (n=2025 babies)**								
Female	438	47·1	315	46·5	199	47·7	952	47·0
Male	485	52·2	361	53·2	218	52·3	1064	52·5
Unknown	7	0·8	2	0·3	0	0·0	9	0·4
**Baby status at birth**								
Baby alive at birth	919	98·8	642	94·7	392	94·0	1953	96·4
Fresh stillbirth	3	0·3	24	3·5	4	1·0	31	1·5
Macerated stillbirth	5	0·5	11	1·6	3	0·7	19	0·9
Unknown/don't know/missing	3	0·3	1	0·1	18	4·3	22	1·1
**Baby status at end of observation period**								
Baby admitted to special care baby unit	124	13·3	14	2·1	53	12·8	191	9·4
Very early infant death (birth to 2 hours postpartum)	15	1·6	17	2·6	12	1·5	44	2·3

Web appendices

Community survey: Physical abuse, verbal abuse, and stigma and discrimination

	Ghana		Guinea		Myanmar		Nigeria		Total	
	n	%	n	%	n	%	n	%	n	%
**Overall sample**	836	31·3	644	24·1	631	23·6	561	21·0	2672	100·0
**Any physical abuse, verbal abuse, or stigma and discrimination***	308	36·8	235	36·5	131	20·8	271	48·3	945	35·4
**Any physical abuse***	52	6·2	124	19·3	21	3·3	90	16·0	287	10·7
Slap	33	4·0	4	0·6	5	0·8	62	11·1	104	3·9
Forceful downward pressure on the abdomen	13	1·6	101	15·7	14	2·2	30	5·4	158	5·9
Pinch	2	0·2	5	0·8	2	0·3	8	1·4	17	0·6
Held down to the bed forcefully	4	0·5	10	1·6	0	0·0	3	0·5	17	0·6
Hit	0	0·0	4	0·6	3	0·5	6	1·1	13	0·5
Punch	0	0·0	8	1·2	0	0·0	4	0·7	12	0·4
Kick	1	0·1	2	0·3	0	0·0	3	0·5	6	0·2
Gag	1	0·1	2	0·3	0	0·0	3	0·5	6	0·2
Tied to the bed	1	0·1	0	0	2	0·3	0	0·0	3	0·1
Other physical abuse	0	0·0	1	0·2	0	0·0	2	0·4	3	0·1
**Any verbal abuse***	284	34·0	173	26·9	116	18·4	248	44·2	821	30·7
Shouted at	187	22·4	111	17·2	75	11·9	160	28·5	533	20·0
Scolded	79	9·5	63	9·8	45	7·1	70	12·5	257	9·6
Threatened with poor outcome for baby	78	9·3	27	4·2	9	1·4	69	12·3	183	6·9
Mocked	40	4·8	42	6·5	6	1·0	45	8·0	133	5·0
Threatened with medical procedure	55	6·6	23	3·6	6	1·0	48	8·6	132	4·9
Insulted	35	4·2	13	2·0	8	1·3	73	13·0	129	4·8
Hissed at	13	1·6	11	1·7	24	3·8	29	5·2	77	2·9
Threatened to withhold care	29	3·5	2	0·3	1	0·2	32	5·7	64	2·4
Negative comments - her sexual activity	19	2·3	5	0·8	6	1·0	22	3·9	52	2·0
Blamed woman for poor outcome	10	1·2	10	1·6	6	1·0	18	3·2	44	1·7
Negative comments - her appearance	11	1·3	6	1·0	1	0·2	9	1·6	27	1·0
Negative comments - baby's appearance	4	0·5	3	0·5	0	0·0	13	2·3	20	0·8
Threatened with physical violence	5	0·6	6	0·9	1	0·2	3	0·5	15	0·6
Other verbal abuse	2	0·2	1	0·2	0	0·0	4	0·7	7	0·3
**Any stigma and discrimination***	31	3·7	9	1·4	11	1·7	28	5·0	79	3·0
Age	17	2·0	1	0·2	6	1·0	4	0·7	28	1·1
Economic circumstances	6	0·7	2	0·3	3	0·5	12	2·1	23	0·9
Race/ethnicity	5	0·6	4	0·6	1	0·2	8	1·4	18	0·7
Level of education/literacy	7	0·8	0	0·0	1	0·2	4	0·7	12	0·5
Marital status	2	0·2	2	0·3	2	0·3	6	1·1	12	0·5
Religion	2	0·2	0	0·0	0·0	0·0	5	0·9	7	0·3
HIV status	2	0·2	1	0·2	0·0	0·0	0	0·0	3	0·1

* Per woman, so subcategories may not total the summary categories if women experienced >1 subcategory

Web appendices

Community survey: Failure to meet professional standards of care, poor rapport between women and providers, health systems

	Ghana		Guinea		Myanmar		Nigeria		Total	
	n	%	n	%	n	%	n	%	n	%
**Failure to meet professional standards**										
Informed consent and confidentiality										
Was the procedure explained and did you agree to the procedure?										
**Caesarean section**	118	14·1	76	11·8	267	42·3	22	3·9	483	18·1
Not explained, did not agree	12	10·2	4	5·3	2	0·78	0	0·0	18	3·7
Not explained, agreed	1-	8·5	2	2·6	19	7·1	1	4·6	32	6·6
Explained, did not agree	2	1·7	0	0·0	0	0·0	0	0·0	2	0·4
Explained and agreed	75	63·6	68	89·5	243	91·0	18	81·8	404	83·6
Don't know/refuse	19	16·1	2	2·6	3	1·1	3	13·6	27	5·6
**Episiotomy (among n=2187 vaginal births)**	92	12·8	52	9·2	250	68·7	132	24·5	526	24·1
Not explained, did not agree	28	30·4	19	36·5	10	4·0	37	28·0	94	17·9
Not explained, agreed	6	6·5	19	36·5	154	61·6	13	9·9	192	36·5
Explained, did not agree	4	4·3	0	0·0	2	0·8	3	2·3	9	1·7
Explained and agreed	42	45·7	12	23·1	73	29·2	65	49·2	192	36·5
Don't know/refuse	12	13·0	2	3·8	11	4·4	14	10·6	39	7·4
**Induction of labor**	125	15·0	3	0·5	173	27·4	48	8·6	349	13·1
Not explained, did not agree	13	10·4	1	33·3	0	0·0	1	2·1	15	4·3
Not explained, agreed	10	8·0	0	0·0	57	32·9	8	16·7	75	21·5
Explained, did not agree	1	0·8	0	0·0	3	1·7	0	0·0	4	1·1
Explained and agreed	84	67·2	2	66·7	111	64·2	38	79·2	235	67·3
Don't know/refuse/missing	17	13·6	0	0·0	2	1·2	1	2·1	20	5·7
Vaginal exams										
Did not have any vaginal exam	28	3·4	55	8·5	126	20·0	18	3·2	227	8·5
Before a vaginal examination, did staff inform woman why a vaginal exam was needed and obtain permission?										
Not informed, permission not obtained	138	17·1	178	30·2	113	22·4	142	26·2	571	23·4
Not informed, permission obtained	40	5·0	12	2·0	21	4·2	78	14·4	151	6·2
Informed, permission not obtained	201	24·9	104	17·7	98	19·4	89	16·4	492	20·1
Informed and permission obtained	409	50·6	293	49·8	268	53·1	227	41·8	1197	49·0
Don't know/refuse	20	2·5	2	0·3	5	1·0	7	1·3	34	1·4
Staff member discussed private health information from vaginal exam so others could hear										
No	733	90·7	511	86·8	430	85·2	331	61·0	2005	82·0
Yes	55	6·8	67	11·4	71	14·1	210	38·7	403	16·5
Refuse/don't know	20	2·5	11	1·9	4	0·8	2	0·4	37	1·5
Vaginal exam conducted privately, so others could not see										
No	236	29·2	292	49·6	131	25·9	365	67·2	1024	41·9
Yes	558	69·1	293	49·8	372	73·7	178	32·8	1401	57·3
Refuse/don't know	14	1·7	4	0·7	2	0·4	0	0·0	20	0·8
General description of experience of vaginal examinations (n=2445 who had VE)										
Comfortable	71	8·8	153	26·0	375	74·3	46	8·5	645	26·4
A little uncomfortable	142	17·6	193	32·8	93	18·4	141	26·0	569	23·3
Quite uncomfortable	217	26·9	125	21·2	21	4·2	144	26·5	507	20·7
Very uncomfortable	371	45·9	115	19·5	11	2·2	203	37·4	700	28·6
Unknown	7	0·9	3	0·5	5	1·0	9	1·7	24	1·0
**Pain relief**										
Woman offered pain relief during time in hospital										
No	511	61·1	456	70·8	90	14·3	471	84·0	1528	57·2
Yes	244	29·2	187	29·0	536	84·9	87	15·5	1054	39·4
Refuse/don't know	81	9·7	1	0·2	5	0·8	3	0·5	90	3·4
If the woman request pain relief, was she given pain relief?										
Not requested, not received	451	54·0	348	54·0	71	11·3	409	72·9	1279	47·9
Not requested, received	205	24·5	80	12·4	374	59·3	96	17·1	755	28·3
Requested, not received	40	4·8	67	10·4	15	2·4	29	5·2	151	5·7
Requested and received	45	5·4	145	22·5	166	26·3	22	3·9	378	14·1

Refuse/don't know	95	11·4	4	0·6	5	0·8	5	0·9	109	4·1
Woman denied pain relief during time in hospital										
No	652	78·0	623	96·7	592	93·8	491	87·5	2358	88·2
Yes	46	5·5	19	3·0	34	5·4	50	8·9	149	5·6
Refuse/don't know	138	16·5	2	0·3	5	0·8	20	3·6	165	6·2
**Neglect and abandonment**										
Was a staff member present when the baby came out? (among n=2187 vaginal births)										
No	24	3·4	2	0·4	2	0·6	16	3·0	44	2·0
Yes	692	96·5	564	99·5	362	99·5	522	96·9	2140	97·9
Refuse/don't know	1	0·1	1	0·2	0	0·0	1	0·2	3	0·1
Waited for long periods of time before attended by health workers										
Agree	281	33·6	75	11·7	139	22·0	92	16·4	587	22·0
Disagree	553	66·2	566	87·9	491	77·8	468	83·4	2078	77·8
Unknown	2	0·2	3	0·5	1	0·2	1	0·2	7	0·3
Felt ignored, neglected, or that presence was a nuisance for health workers or staff										
Agree	137	16·5	72	11·3	121	19·4	104	18·6	434	16·4
Disagree	696	83·6	565	88·7	502	80·6	455	81·4	2218	83·6
**Poor rapport**										
Communication										
Language interpretation										
Did not need an interpreter	824	98·6	629	97·7	630	99·8	559	99·6	2642	98·9
Needed an interpreter and had one present	8	1·0	12	1·9	0	0·0	0	0·0	20	0·7
Needed an interpreter and did not have one present	2	0·2	3	0·5	0	0·0	2	0·4	7	0·3
Don't know/refuse	2	0·2	0	0·0	1	0·2	0	0·0	3	0·1
Health workers or staff listened and responded to my concerns										
Agree	713	85·3	528	82·0	400	63·4	487	86·8	2128	79·6
Disagree	105	12·6	113	17·6	200	31·7	59	10·5	477	17·9
Unknown	18	2·2	3	0·5	31	4·9	15	2·7	67	2·5
Supportive care										
Allowed to have a labor companion during labor and birth?										
No	486	58·1	336	52·2	5	0·8	373	66·5	1200	44·9
Yes	342	40·9	305	47·4	626	99·2	187	33·3	1460	54·6
Refuse/don't know	8	1·0	3	0·5	0	0·0	1	0·2	12	0·4
Had a labor companion present at any point										
No	437	52·3	560	87·0	1	0·2	320	57·0	1318	49·3
Yes	395	47·3	82	12·7	629	99·7	240	42·8	1346	50·4
Refuse/don't know	4	0·5	2	0·3	1	0·2	1	0·2	8	0·3
Supportive care										
Autonomy										
Easy access to water or oral fluids (n=2187 women with vaginal birth)										
No	89	12·4	74	13·1	50	13·7	214	39·7	427	19·5
Yes	619	86·3	489	86·2	311	85·4	322	59·7	1741	79·6
Refuse/don't know	9	1·3	4	0·7	3	0·8	3	0·6	19	0·9
Allowed to eat (n=2187) women with vaginal birth)										
No	298	41·6	82	14·5	1	0·3	327	60·7	708	32·4
Yes	398	55·5	478	84·3	362	99·5	200	37·1	1438	65·8
Refuse/don't know	21	2·9	7	1·2	1	0·3	12	2·2	41	1·9
Woman told she could mobilize during labor, and if she mobilized during labor										
Not told, did not mobilize	582	69·6	29	4·5	202	32·0	443	79·0	1256	47·0
Told, did not mobilize	42	5·0	15	2·3	34	5·4	13	2·3	104	3·9
Not told, mobilized	83	9·9	16	2·5	81	12·8	67	11·9	247	9·2
Told and mobilized	118	14·1	584	90·7	306	48·5	34	6·1	1042	39·0
Don't know/refuse	11	1·3	0	0·0	8	1·3	4	0·7	23	0·9
Preferred birth position										
No	810	96.9	517	80.3	512	81.1	526	93.8	2365	88.5
Yes	19	2.3	126	19.6	96	15.2	32	5.7	273	10.2
Unknown	7	0.8	1	0.2	23	3.7	3	0.5	34	1.2

Actual birth position (for women with vaginal birth, n=2187)										
Dorsal/supine	190	26·5	565	99·7	18	5·0	473	87·8	1246	57·0
Lithotomy	510	71·1	1	0·2	338	92·9	57	10·6	906	41·4
On all fours	1	0·1	0	0·0	1	0·3	0	0·0	2	0·1
Squatting or sitting	3	0·4	1	0·2	1	0·3	5	0·9	10	0·5
Lying on her side	6	0·8	0	0·0	3	0·8	1	0·2	10	0·5
Other/unknown	7	1·0	0	0·0	3	0·8	3	0·6	13	0·6
Woman or baby detained in hospital due to inability to pay hospital bills										
No	723	86·5	583	90·5	624	98·9	529	94·3	2459	92·0
Yes	40	4·8	56	8·7	7	1·1	30	5·4	133	5·0
Refuse/don't know	73	8·7	5	0·8	0	0·0	2	0·4	80	3·0
Curtains, partitions or other privacy measures used										
No	65	7·8	339	52·6	330	52·3	468	83·4	1202	45·0
Yes	763	91·3	299	46·4	296	46·9	93	16·6	1451	54·3
Refuse/don't know	8	1·0	6	0·9	5	0·8	0	0·0	19	0·7
Staff suggested or asked for a bribe, informal payment, or gift										
No	726	86·8	320	49·7	370	58·6	490	87·3	1906	71·3
Yes	104	12·4	306	47·5	255	40·4	60	10·7	725	27·1
Refuse/don't know	6	0·7	18	2·8	6	1·0	11	2·0	41	1·5
Asked to clean up own blood, urine, feces or amniotic fluid after birth										
No	827	98·9	636	98·8	528	83·7	554	98·8	2545	95·2
Yes	8	1·0	6	0·9	99	15·7	5	0·9	118	4·4
Refuse/don't know	1	0·1	2	0·3	4	0·6	2	0·4	9	0·3

Web appendices

Comparison of our study to other mistreatment studies using labor observation and community-based surveys

Labor observations

Other studies exploring the prevalence of mistreatment during childbirth have used labor observations, but different periods of interest, measurement tools and outcomes, which complicates comparison. Seven studies conducted in LMICs (Ethiopia, India, Kenya, Madagascar, Malawi, Rwanda and Tanzania) have used direct observations of labor and childbirth to measure how women are treated during childbirth,[1-7] based on criteria in Bowser and Hill's landscape analysis.[8,9] The studies had variable periods of observation: admission to immediate postpartum and newborn care,[3,5-7] second-stage of labor to two-hours postpartum,[1] intermittently during admission, first-stage of labor, and immediate postpartum, and continuously during the second- and third-stage of labor,[2] and admission to one-hour postpartum.[4] Continuous coverage of the observations also varied, including sixteen-hours per day in two eight-hour shifts,[1,5] 24-hour coverage in three shifts,[3,4,6] and each observer conducting five observations per day.[2] Most observers had a clinical background (nurse, midwife, doctor), but were not employed at the study site,[1-5,7] In our study, 14·0% of women experienced physical abuse and 37·8% of women experienced verbal abuse. Other labor observations conducted in Ethiopia, Malawi and Kenya reported lower levels of physical abuse (0·2% to 9·0%) and verbal abuse (1·9% to 18·1%),[1,2,5] and similar levels of providers not explaining procedures to women (17·1% to 77·0%),[2,5] and not informing women what will happen during a vaginal examination (20·5% to 81·0%).[2,3] About one quarter of women in Tanzania[3] and one-third of women in Kenya[10] had insufficient curtains or partitions during labor and childbirth, compared to almost half in our study. In Malawi, 88% of women did not have a labor companion present at the time of birth,[2] similar to our study.

Postpartum surveys

Fifteen existing studies have measured women-reported experiences of mistreatment during childbirth using survey methodology in Brazil, India, Ethiopia, Tanzania, India, Pakistan, Kenya, Peru and Nigeria.[3,6,7,10-21] Eight of these studies used community-based interviews,[7,11-16,22] eight used facility-based interviews[3,6,10,17,18,20-22] (Kruk et al used both community- and facility-based interviews). Six of the studies using facility-based interviews were conducted during the postpartum period prior to discharge,[3,6,10,17,18,22] and two were conducted at immunization clinic at around 6 weeks postpartum.[20,21] Among studies using community-based surveys, the timing of the interview varied substantially including up to: 2-4 weeks postpartum,[7] 8 weeks postpartum,[15] 5-10 weeks postpartum,[22] 12 weeks postpartum,[11] one year postpartum,[12,16] two years postpartum,[13] and five years postpartum.[14] Four existing studies measured women-reported experiences of mistreatment during childbirth within a similar time period (2-12 weeks postpartum) and in a community setting.[7,11,15,22] In our study, 10·7% reported any physical abuse and 30·7% reported any verbal abuse. In Uttar Pradesh, less than 1% of women reported being beaten or slapped and 2·6% of women reported providers using bad or abusive language.[7] In Gujrat, Pakistan, 19·2% of women reported physical abuse. In Pelotas, Brazil, 5% of women reported physical abuse and 10% of women reported verbal abuse.[11] In Tanga, Tanzania, 5·1% of women reported physical abuse and 18·9% of women reported non-dignified care.[18] Afulani and colleagues used facility-based surveys to

measure person-centered maternity care in Ghana, India and Kenya, and found that approximately two-thirds of women reported that providers did not explain the purpose of examinations or procedures.[23]

The timing and location of the interviews may impact estimates of mistreatment in several ways. Facility-based interviews (exit or at immunization clinic) may underestimate mistreatment if women are concerned that disclosure may impact care received for her or her baby, or due to courtesy or social desirability bias. Women's recall may affect community-based interviews conducted longer after her birth (e.g. more than 6-months postpartum). For example, Kruk and colleagues used exit and community-based follow-up interviews with the same women and found that women reported higher rates of any abusive or disrespectful treatment during childbirth in the follow-up (28·2%) compared to the exit (19·5%) interviews.[22]

References

1. Sheferaw ED, Bazant E, Gibson H, et al. Respectful maternity care in Ethiopian public health facilities. *Reprod Health* 2017; **14**(1): 60.
2. Sethi R, Gupta S, Oseni L, Mtimuni A, Rashidi T, Kachale F. The prevalence of disrespect and abuse during facility-based maternity care in Malawi: evidence from direct observations of labor and delivery. *Reprod Health* 2017; **14**(1): 111.
3. Sando D, Ratcliffe H, McDonald K, et al. The prevalence of disrespect and abuse during facility-based childbirth in urban Tanzania. *BMC Pregnancy Childbirth* 2016; **16**: 236.
4. Abuya T, Ndwiga C, Ritter J, et al. The effect of a multi-component intervention on disrespect and abuse during childbirth in Kenya. *BMC Pregnancy Childbirth* 2015; **15**: 224.
5. Rosen HE, Lynam PF, Carr C, et al. Direct observation of respectful maternity care in five countries: a cross-sectional study of health facilities in East and Southern Africa. *BMC Pregnancy Childbirth* 2015; **15**: 306.
6. Banks KP, Karim AM, Ratcliffe HL, Betemariam W, Langer A. Jeopardizing quality at the frontline of healthcare: prevalence and risk factors for disrespect and abuse during facility-based childbirth in Ethiopia. *Health Policy Plan* 2018; **33**(3): 317-27.
7. Dey A, Shakya HB, Chandurkar D, et al. Discordance in self-report and observation data on mistreatment of women by providers during childbirth in Uttar Pradesh, India. *Reprod Health* 2017; **14**(1): 149.
8. Bowser D, Hill K. Exploring Evidence for Disrespect and Abuse in Facility-Based Childbirth: Report of a Landscape Analysis: USAID, 2010.
9. White Ribbon Alliance. Respectful Maternity Care: The Universal Rights of Childbearing Women: White Ribbon Alliance, October 2011.
10. Abuya T, Warren CE, Miller N, et al. Exploring the prevalence of disrespect and abuse during childbirth in Kenya. *PLoS ONE* 2015; **10**(4).
11. Mesenburg MA, Victora CG, Jacob Serruya S, et al. Disrespect and abuse of women during the process of childbirth in the 2015 Pelotas birth cohort. *Reprod Health* 2018; **15**(1): 54.
12. Wassihun B, Deribe L, Worede N, Gultie T. Prevalence of disrespect and abuse of women during child birth and associated factors in Bahir Dar town, Ethiopia. *Epidemiology and health* 2018; **40**: e2018029.
13. Bishanga DR, Massenga J, Mwanamsangu AH, et al. Women's Experience of Facility-Based Childbirth Care and Receipt of an Early Postnatal Check for Herself and Her Newborn in Northwestern Tanzania. *Int J Environ Res Public Health* 2019; **16**(3).
14. Diamond-Smith N, Sudhinaraset M, Melo J, Murthy N. The relationship between women's experiences of mistreatment at facilities during childbirth, types of support received and person providing the support in Lucknow, India. *Midwifery* 2016; **40**: 114-23.
15. Azhar Z, Oyebode O, Masud H. Disrespect and abuse during childbirth in district Gujrat, Pakistan: A quest for respectful maternity care. *PLoS One* 2018; **13**(7): e0200318.
16. Hameed W, Avan BI. Women's experiences of mistreatment during childbirth: A comparative view of home- and facility-based births in Pakistan. *PLoS One* 2018; **13**(3): e0194601.
17. Asefa A, Bekele D. Status of respectful and non-abusive care during facility-based childbirth in a hospital and health centers in Addis Ababa, Ethiopia. *Reprod Health* 2015; **12**: 33.
18. Montesinos-Segura R, Urrunaga-Pastor D, Mendoza-Chuctaya G, et al. Disrespect and abuse during childbirth in fourteen hospitals in nine cities of Peru. *Int J Gynaecol Obstet* 2018; **140**(2): 184-90.
19. Kujawski S, Mbaruku G, Freedman LP, Ramsey K, Moyo W, Kruk ME. Association Between Disrespect and Abuse During Childbirth and Women's Confidence in Health Facilities in Tanzania. *Maternal and child health journal* 2015; **19**(10): 2243-50.
20. Okafor I, Ugwu E, Obi SN. Disrespect and abuse during facility-based childbirth in a low-income country. *Int J Gynaecol Obstet* 2015; **128**(2): 110-3.
21. Ijadunola MY, Olotu EA, Oyedun OO, et al. Lifting the veil on disrespect and abuse in facility-based child birth care: findings from South West Nigeria. *BMC Pregnancy Childbirth* 2019; **19**(1): 39.
22. Kruk ME, Kujawski S, Mbaruku G, Ramsey K, Moyo W, Freedman LP. Disrespectful and abusive treatment during facility delivery in Tanzania: a facility and community survey. *Health Policy and Planning* 2014.
23. Afulani PA, Phillips B, Aborigo RA, Moyer CA. Person-centred maternity care in low-income and middle-income countries: analysis of data from Kenya, Ghana, and India. *The Lancet Global Health* 2019; **7**(1): e96-e109.

Web appendices

Labor observation: Assessing the presence of the Hawthorne effect, by country and month of recruitment, based on any experience of physical abuse, verbal abuse, or stigma and discrimination.

Observations may be limited by the risk of the Hawthorne effect as the presence of observers may alter the provider's behavior and result in underestimation of mistreatment. We explored this by study site, country and month of recruitment and found no evidence of the presence of the Hawthorne effect in the following tables. We note that the analysis below is limited because it was not possible to record the rate of mistreatment events before data collection started – ideally, this would have occurred in the month prior to the project start date. Therefore, it is possible that having an observer present from the start of data collection (month 1) may have influenced staff behavior. We hypothesize that the risk of influencing staff behavior may be low, because we did not conduct any quality improvement or quality of care training with providers as part of the study implementation, but are unable to prove this hypothesis from our data.

**NIGERIA**					
Any mistreatment (%)					
	Facility 1	Facility 2	Facility 3	Nigeria Overall	P-value
Month 1	45%	63%	60%	55%	NS
Month 1: Labor ward admit	n=42	n=22	n=37		
Month 2	62%	100%	62%	63%	NS
Month 2: Labor ward admit	n=83	n=6	n=52		
Month 3	88%	62%	72%	79%	NS
Month 3: Labor ward admit	n=44	n=13	n=11		
Month 4	71%	85%	0%	81%	NS
Month 4: Labor ward admit	n=14	n=54	n=1		
**GUINEA**					
Any mistreatment (%)					
	Facility 1	Facility 2	Facility 3	Guinea Overall	P-value
Month 1	41%	46%	47%	45%	NS
Month 1: Labor ward admit	n=44	n=41	n=36		
Month 2	49%	34%	45%	44%	NS
Month 2: Labor ward admit	n=111	n=70	n=80		
Month 3	36%	33%	44%	38%	NS
Month 3: Labor ward admit	n=111	n=60	n=50		
Month 4	19%	35%	36%	31%	NS
Month 4: Labor ward admit	n=57	n=51	n=69		
**GHANA**					
Any mistreatment (%)					
	Facility 1	Facility 2	Facility 3	Ghana Overall	P-value
Month 1	34%	31%	41%	36%	NS
Month 1: Labor ward admit	n=125	n=77	n=129		
Month 2	40%	37%	30%	35%	NS
Month 2: Labor ward admit	n=121	n=87	n=152		
Month 3	32%	14%	39%	25%	NS
Month 3: Labor ward admit	n=59	n=79	n=33		
Month 4	0%	7%	N/A	7%	N/A
Month 4: Labor ward admit	n=3	n=57	N/A		

Web appendices

OVERALL BY COUNTRY			
	Any mistreatment (%)		
	Nigeria	Ghana	Guinea
Month 1	55%	36%	45%
Month 2	63%	35%	44%
Month 3	79%	25%	38%
Month 4	81%	7%	31%
Month 5	78%	-	-
Month 6	75%	-	-

NS: Not significant
